# Control of myeloid-derived suppressor cell dynamics potentiates vaccine protection in multiple mouse models of *Trypanosoma cruzi* infection

**DOI:** 10.3389/fimmu.2024.1484290

**Published:** 2024-11-01

**Authors:** Eliana Borgna, Estefanía Prochetto, Juan Cruz Gamba, Elba Mónica Vermeulen, Carolina Verónica Poncini, Pamela Cribb, Ana Rosa Pérez, Iván Marcipar, Florencia Belén González, Gabriel Cabrera

**Affiliations:** ^1^ Facultad de Bioquímica y Ciencias Biológicas, Universidad Nacional del Litoral, Santa Fe, Argentina; ^2^ Facultad de Ciencias Médicas, Universidad Nacional del Litoral, Santa Fe, Argentina; ^3^ Laboratorio de Células Presentadoras de Antígeno y Respuesta Inflamatoria, Instituto de Medicina Experimental (IMEX-CONICET), Academia Nacional de Medicina, Buenos Aires, Argentina; ^4^ Laboratorio de Inmunología Celular e Inmunopatología de Infecciones, IMPaM UBA-CONICET, Departamento de Microbiología, Parasitología e Inmunología, Facultad de Medicina, Universidad de Buenos Aires, Buenos Aires, Argentina; ^5^ Instituto de Biología Molecular y Celular de Rosario (IBR-CONICET), Universidad Nacional de Rosario, Santa Fe, Argentina; ^6^ Instituto de Inmunología Clínica y Experimental de Rosario (IDICER-CONICET), and Facultad de Ciencias Médicas, Universidad Nacional de Rosario, Santa Fe, Argentina; ^7^ Centro de Investigación y Producción de Reactivos Biológicos (CIPReB), Facultad de Ciencias Médicas, Universidad Nacional de Rosario, Santa Fe, Argentina

**Keywords:** *Trypanosoma cruzi*, vaccine, myeloid-derived suppressor cells, effector response, 5-fluorouracil, Chagas disease, dendritic cells, adjuvant

## Abstract

To date, there is no licensed vaccine against the protozoan parasite *Trypanosoma cruzi* (*T. cruzi)*, the etiological agent of Chagas Disease. *T. cruzi* has evolved numerous mechanisms to evade and manipulate the host immune system. Among the subversive strategies employed by the parasite, marked increases in CD11b+ Gr-1+ myeloid-derived suppressor cells (MDSCs) in several organs have been described. We have reported that CD11b+ Gr-1+ cells are involved not only during infection but also after immunization with a trans-sialidase fragment (TSf) adjuvanted with a cage-like particle adjuvant (ISPA). Thus, the aim of this work was to gain control over the involvement of MDSCs during immunization to potentiate a vaccine candidate with protective capacity in multiple mouse models of *T. cruzi* infection. Here, we show that the Gr-1+ cells that increase during TSf-ISPA immunization have suppressive capacity over bone marrow-derived dendritic cells and CD4+ lymphocytes. Protocols using one or two doses of 5-fluorouracil (5FU) were employed to deplete and control MDSC dynamics during immunization. The protocol based on two doses of 5FU (double 5FU TSf-ISPA) was more successful in controlling MDSCs during immunization and triggered a higher immune effector response, as evidenced by increased numbers of CD4+, CD4+CD44+, CD8+, CD8+CD44+, CD11c+, and CD11c+CD8α+ cells in the spleen and lymph nodes of double 5FU TSf-ISPA mice as compared to 5FU-TSf-ISPA mice. In line with these results, the protective capacity of the double 5FU TSf-ISPA protocol was higher compared to the 5FU-TSf-ISPA protocol against high lethal doses of intraperitoneal infection with the Tulahuen *T. cruzi* strain. When cross-protective capacity was analyzed, the optimized protocol based on double 5FU TSf-ISPA conferred protection in several preclinical models using different discrete typing units (DTU VI and DTU I), different mouse strains (BALB/c and C57BL/6), different parasite doses (1000 to 20000), and routes of administration (intraperitoneal and intradermal). Developing vaccines that are currently lacking may require new strategies to further potentiate vaccine candidates. Results reported herein provide evidence that rational control of cells from the regulatory arm of the immune system could enhance a vaccine candidate with cross-protective capacity in multiple mouse models of *T. cruzi* infection.

## Introduction

Despite significant progress in vaccine development, there are still many pathogens for which vaccines have not been developed yet (1). Some characteristics that have been suggested to hinder vaccine development include, in many cases, complex life cycles, genetic diversity (2, 3) and the ability to subvert the host regulatory immune system (4–8). *Trypanosoma cruzi (T. cruzi)*, the etiological agent of Chagas disease, fulfills these three requirements, and there is no prophylactic nor therapeutic vaccine available.

*T. cruzi* strains have been classified into seven discrete typing units (DTUs): TcI to TcVI, plus Tcbat (9). To date, very few studies have assessed the cross-protective capacity of vaccine candidates against *T. cruzi*. 10 studied the protective immunity elicited by vaccination with ASP-2 and trans-sialidase recombinant vaccines, supporting the fact that protection was strain-specific after infection with the Colombian and Colombia strains. In addition, 11 analyzed whether a chimeric protein based on ASP-2 and TS regions could serve as a vaccine candidate against the CL-Brener (TcVI) and Be-78 (TcII) strains of *T. cruzi* (3, 10, 11). It has been suggested that the antigen used as immunogen may be critical to elicit protection against different DTUs (2, 12). In this sense, trans-sialidase (TS) antigens are among the parasite proteins suggested to have cross-protective potential (12), and numerous studies focusing on TS have provided evidence that this protein is a promising candidate for the development of a vaccine against *T. cruzi* (13, 14). In addition, the fact that humans do not have orthologs of TS genes also highlights the potential use of TS as a vaccine candidate (14).

The TS genes are part of a large superfamily divided into eight groups (**Supplementary Table 1**) (15, 16). Group I of TS includes genes that encode a functional enzyme capable of transferring sialic acid molecules from host glycoconjugates to acceptor molecules on the surface of the parasite or the host (15). This mechanism has been related to several pathways of subverting the host immune system (17, 18), which may constitute an obstacle to the development of a successful vaccine against *T. cruzi*. In addition, regarding the capacity of *T. cruzi* to manipulate the host immune system, numerous studies have described that acute infection causes increases of various immune populations with regulatory capacity, including a notable expansion of myeloid-derived suppressor cells (MDSCs) in the spleen, liver and cardiac tissue (19–23).

MDSCs have been described as a heterogeneous immature population from myeloid origin, whose main hallmark is the ability to suppress immune responses. In mice, they are typically characterized by co-expression of CD11b and Gr-1 markers, with two main subsets: G-MDSCs (CD11b+ Ly6G+ Ly6Clow/+, also termed granulocytic-MDSCs or polymorphonuclear PMN-MDSCs) and M-MDSCs (CD11b+ Ly6G- Ly6C+, also known as monocytic-MDSCs) (24). Several reports have shown the suppressive capacity of the CD11b+ Gr-1+ cells that increase during acute *T. cruzi* infection (19, 21, 25). Moreover, it has been suggested that MDSCs may play a role in attenuating the inflammatory response, favoring the survival of the host, but also decreasing the effector response and allowing the persistence of the parasite (21). While their involvement in *T. cruzi* infection is well-documented, emerging evidence suggests that MDSCs may also play a role during the immunization phase against *T. cruzi* (25, 26), a characteristic that has also been reported during vaccinations against many other pathogens (27).

We have previously shown that a formulation based on a trans-sialidase fragment (TSf) adjuvanted with a cage-like particle (ISPA) was able to confer protection against T. *cruzi* infection (26, 28). This formulation decreased parasitemia and increased survival in a model of BALB/c mice intraperitoneally (i.p.) challenged with trypomastigotes of the Tulahuen *T. cruzi* strain. The TSf-ISPA immunization generated an effector humoral and cellular immune response that contributed to protection but also affected CD11b+ Gr-1+ cells during both the immunization and infection phases. Increased levels of CD11b+ Gr-1+ splenocytes were observed in vaccinated mice during immunization, whereas after infection, immunized mice showed a significant decrease in MDSCs compared to control PBS-treated and infected mice, which exhibited very high levels of MDSCs (26).

According to these results, further studies were conducted to gain insight into the physiological role of MDSCs during *T. cruzi* infection in vaccinated mice. Since 5-fluorouracil (5FU) (50 mg/kg) has been used to selectively deplete MDSCs in the context of cancer and other settings (29), including *T. cruzi* infection (21, 22), vaccinated mice were depleted of MDSCs with 5FU at day 15 post-infection, and alterations in several populations were analyzed in the spleen. Depletion of MDSCs during infection of TSf-ISPA vaccinated mice reshaped the immune response, including the generation of a stronger CD8 response, an increase in the absolute number of Foxp3+ regulatory T cells (Tregs), and an influence on the expression of costimulatory molecules by dendritic cells (DCs) (25). Although evidence supports that MDSCs can induce Tregs, mainly in cancer settings, a potential role in suppressing Tregs has also been described (30–32). These results suggest that even when the TSf-ISPA vaccine candidate decreased MDSC levels in the spleen during infection, the remaining MDSCs still had the capacity to influence and shape the immune response, which is expected to control *T. cruzi* infection.

Considering our results, which show that CD11b+ Gr-1+ cells are significantly involved both during immunization and after *T. cruzi* infection (25, 26), we envisioned that these cells could be targeted to improve the protective capacity of the TSf-ISPA vaccine candidate. As a first approach, 5FU was administered one day before each dose of the TSf-ISPA vaccine, resulting in a new formulation called the 5FU-TSf-ISPA vaccine. After challenging mice with a high lethal dose of trypomastigotes of the Tulahuen strain, the results showed that depleting CD11b+ Gr-1+ cells during immunization significantly increased the protective capacity of our vaccine candidate (25).

After decades of research, the enhancement of a vaccine candidate against *T. cruzi* remains elusive. This parasite generally establishes chronic infection even in vaccinated mice, likely because vaccines cannot neutralize all the mechanisms the parasite employs to evade, interfere with, and manipulate the immune system. These mechanisms include escaping complement-mediated lysis (33), delaying the CD8 response (34, 35), inducing a non-specific B cell response (36, 37), promoting the induction of tolerogenic dendritic cells (38–40), triggering the apoptosis of T lymphocytes (41), causing alterations in Tregs (8, 42–45), and inducing a significant expansion of MDSCs (19–21, 46), among other strategies reported. In such a scenario, the usual strategy to target only the effector response may not be sufficient, and considering the role of immunoregulatory populations such as MDSCs may become an alternative approach to complement and potentiate vaccine candidates. Therefore, given previous evidence that immunization with TSf adjuvanted with ISPA generated significant protection against *T. cruzi* infection, but also led to an increase in CD11b+ Gr-1+ cells resembling MDSCs — potentially hindering further enhancement of vaccine efficacy — the aim of this study was to control the involvement of CD11b+ Gr-1+ cells in the immune response triggered by a TS-based vaccine, to further optimize a protocol with protective capacity in multiple mouse models of *T. cruzi* infection.

## Materials and methods

### Mice and parasites

BALB/c and C57BL/6 female mice (6-8 weeks old) used in all experimental procedures were obtained from the Centro de Medicina Comparada (CMC) of Universidad Nacional del Litoral (UNL), Argentina. All protocols for animal studies were approved by the Advisory Committee of Ethics and Research Safety (CAESI) of the Facultad de Bioquímica y Ciencias Biológicas, UNL, Argentina, according to international ethical guidelines for biomedical research involving animals (CAESI 2019-7/19).

Tulahuen blood trypomastigotes were obtained from the blood of Cb1-infected mice, as previously reported (25, 43). Cb1 is an outbreeding albino strain that is bred in the animal facilities of the Centro de Investigación y Producción de Reactivos Biológicos (CIPReB-FCM-UNR), following protocols for animal studies approved by the Institutional Bioethics and Biosecurity Committees (Resolution N 3913/2008). Dm28c trypomastigotes were obtained *in vitro* from VERO cells cultured in complete RPMI 1640 medium (GibcoTM) supplemented with 10% fetal bovine serum (Internegocios SA) and 2% penicillin (100 mg/mL) and streptomycin (100 U/mL) (Gibco TM).

Parasites from RA strain (47) were maintained by weekly intraperitoneal inoculation of three weeks-old male CF1 mice (1 × 10^5^ parasites/mouse). RA bloodstream forms (Tp) were obtained from whole blood at the peak of parasitemia 7 days post-infection. RA strain was exclusively used to infect BALB/c male mice according to protocols CD N° 04/2015 approved by the University of Buenos Aires´s Institutional Committee for the Care and Use of Laboratory Animals (CICUAL) in accordance with the Council for International Organizations of Medical Sciences (CIOMS) and International Council for Laboratory Animal Science (ICLAS) international ethical guidelines for biomedical research involving animals.

BALB/c male mice were immunized and challenged with RA parasites as described in **Supplementary Figure 7**.

### Immunization schedules

BALB/c mice (n=5-10/group) were immunized with three subcutaneous doses, one every two weeks, containing 10 µg of a trans-sialidase antigen (TSf), (genBank AJ276679.1), prepared as previously described (26), with 3 µl of ISPA as adjuvant (group TSf-ISPA). ISPA is a cage-like particle adjuvant prepared as previously described, using dipalmitoylphosphatidylcholine (DPPC), cholesterol (CHOL), stearylamine (STEA), tocopherol (TOCOP), and Quillaja Saponaria (QuilA) (28).

The 5FU (Sigma Aldrich) was administered at a dose of 50mg/kg, a dosage previously described to selectively deplete MDSCs in several mice studies (29, 48) and even during *T. cruzi* infection (21, 22). BALB/c or C57BL/6 mice were treated with 5FU one day before TSf-ISPA vaccine (group 5FU TSf-ISPA) or both before each dose of TSf-ISPA vaccine and 7 days after each TSf-ISPA immunization, in a protocol termed double 5FU TSf-ISPA (group double 5FU TSf-ISPA). (According to the experiment, when the double 5FU TSf-ISPA group of mice was sacrificed two days or seven days after the third immunization dose, they did, in fact, receive the 5FU dose at day 7 post immunization during the two initial immunizations but not after the third immunization (**Supplementary Figure 1**).

Control groups were immunized with phosphate-buffered saline (PBS) solution, following the same protocol (group PBS).

Several independent groups have already shown that 5FU administration at the dose employed does not affect other immune populations (22, 29, 48).

Additionally, we have already reported that TSf administration alone or ISPA administration alone does not generate protective capacity against *T. cruzi* (26).

### Humoral response and delayed-type hypersensitivity (DTH)

Mouse blood was obtained following the method previously described (49). Subsequently, the level of plasmatic IgG total antibodies (IgGt) anti-TSf was evaluated by ELISA 7 days after the third TSf-ISPA dose, as we previously described (26). Delayed-type hypersensitivity (DTH) test was performed 5 days after the third TSf-ISPA dose by intradermal (i.d.) challenge with 5 μg of TSf in the right hind footpad, as we previously described (26).

### Suppression assay

Gr-1+ cells were purified by magnetic cell sorting from spleens of immunized BALB/c mice using the Myeloid-Derived Suppressor Cell Isolation Kit (Miltenyi Biotec), according to the manufacturer’s instructions. On day 7 post-immunization (post-imm.), TSf-ISPA immunized mice were sacrificed, spleens were aseptically removed, and splenocytes were harvested as we previously described (25). After purification, the purity of Gr-1+ purified cells was higher than 90%, as evaluated by flow cytometry using an anti-Gr-1 antibody (BD-Biosciences).

Bone-marrow-derived dendritic cells (BMDCs) were obtained from healthy BALB/c mice. Briefly, femurs were removed from mice, and bone marrow cells were collected by rinsing RPMI 1640 (GibcoTM) medium inside the bone. The purified cells were cultured in a specific medium for dendritic cell differentiation: RPMI 1640 medium (Gibco TM), 10% fetal bovine serum (Internegocios SA), 2% penicillin (100 mg/mL), streptomycin (100 U/mL) (GibcoTM), and 30% J588-GM cell line supernatant (50). Cells were seeded in 24-well plates (Nunc) (0.75x10^6^ cells/well) at 37°C and 5% CO_2_. On days 3 and 5, fresh medium was added. On day 8, BMDCs were harvested and used for flow cytometry analysis and cultures.

The acquisition of cells compatible with Bone Marrow-Derived Dendritic Cells (BMDCs) was controlled by assessing the expression of costimulatory molecules in CD11c+ cells stimulated with Lipopolysaccharide (LPS) from *Escherichia coli* (ThermoFisher). For this purpose, 1x10^5^ BMDCs were cultured in 96 well plates (Nunc) in RPMI 1640 medium (Gibco TM), 10% fetal bovine serum (Internegocios SA), 2% penicillin (100 mg/mL), streptomycin (100 U/mL) for 24 h in the presence or absence of LPS (1µg/ml). Flow cytometry using anti-CD11c (Miltenyi Biotec), anti-CD80 (BD-Biosciences), and anti-MHCII (Miltenyi Biotec) antibodies in appropriate combinations of fluorophores confirmed the obtention of CD11c+ cells with the capacity to increase the expression of costimulatory markers in the presence of LPS, as previously described by (40).

Spleen cells from untreated BALB/c mice were labeled with 10 μM of 5,6-carboxifluorescein diacetate succinimidyl ester (CFSE) (Molecular probes, Eugene, OR), following the manufacturer’s instructions. The CFSE label was verified by flow cytometry.

For the suppression assay, 1x10^5^ Gr-1+ cells were cultured with 1x10^5^ BMDCs alone or in the additional presence of 1x10^5^ CFSE-stained splenocytes in 96-well plates (Nunc) in supplemented RPMI medium (RPMI 1640 medium (GibcoTm), 10% fetal bovine serum (Internegocios), 2% penicillin (100 mg/mL), streptomycin (100 U/mL) (GibcoTM). LPS (1µg/ml, ThermoFisher) was added to cultures containing BMDCs and MDSCs. LPS (1µg/ml, ThermoFisher) plus concanavalin A (ConA, Sigma) (5 µg/ml) were added to wells containing BMDCs, MDSCs, and CFSE-stained splenocytes. To complete the total number of cells per well, BMDCs were added instead of MDSCs in the control cultures without purified Gr-1+ cells. After 72 hours at 37°C and 5% CO_2_, cells were harvested, and the suppressor activity of MDSCs was evaluated by flow cytometry using anti-CD11c (Miltenyi Biotec), anti-CD80 (BD-Biosciences), anti-MHCII (Miltenyi Biotec), and anti-CD4 (BD-Biosciences) antibodies conjugated to fluorophores. Flow cytometry was performed in a similar manner as described below.

### Flow cytometry and cell culture

The kinetic analysis of the CD11b+ Gr-1+ MDSC population was performed by flow cytometry. Mice treated with TSf-ISPA, 5FU TSf-ISPA, and double 5FU TSf-ISPA were sacrificed on days 2, 7, and 15 post-third TSf-ISPA dose, and spleen cells were obtained and counted as previously described (25). Additionally, on day 7 post-imm., lymph nodes were also collected.

For flow cytometry, 1x10^6^ cells were first incubated with anti-FcγIII/II receptor antibody (Biolegends), and then stained for 40 minutes at 4°C with appropriate combinations of the following antibodies conjugated to fluorophores from BD-Biosciences: anti-CD11b, anti-Gr-1, anti-Ly6G, anti-Ly6C, anti-CD4, anti-CD8α, anti-CD44, and anti-CD11c. Samples were acquired on a BD AccuriTM C6 plus (BD-Biosciences) cytometer, and data analysis was performed using FlowJo software.

After *T. cruzi* infection, analysis of the MDSC subtypes (G-MDSCs and M-MDSCs) was assessed. Mice immunized with 5FU TSf-ISPA or double 5FU TSf-ISPA were infected with *T. cruzi* 15 days after the last immunization dose and were sacrificed at day 21 post-infection. Splenocytes were harvested as previously described (25), and MDSC subtypes were studied by flow cytometry. PBS-treated mice were also infected, but in many cases did not survive until day 21 post-infection.

Splenocytes from mice sacrificed at day 7 post-immunization were also used for cell culture. After red blood cell lysis with NH_4_Cl, splenocytes were re-suspended in RPMI 1640 medium (Gibco), supplemented with 10% fetal bovine serum (FBS), 100 U/mL penicillin, 100 µg/mL streptomycin (Gibco), and 0.4 mM 2-mercaptoethanol. Cells (0.5 × 10^6^ cells/mL) were cultured in 48-well plates (Nunc) in supplemented RPMI. Splenocytes were stimulated with 5 µg/mL TSf. After 72 hours at 37°C and 5% CO_2_, cells were incubated with monensin (BD-Pharmingen) according to the manufacturer’s instructions. Four hours later, supernatants were collected, and cells were washed twice with PBS, incubated with anti-FcγRIII/II receptor antibody for 30 minutes, and stained with anti-CD8 FITC and anti-CD4 APC for 30 minutes. Cells were then washed and stained for intracellular IFN-γ using PE-labeled anti-IFN-γ antibody with the BD-Pharmingen intracellular staining kit, according to the manufacturer’s instructions. Samples were acquired on an Accuri C6 Plus flow cytometer (BD-Biosciences) and analyzed using FlowJo software.

Additionally, IFN-γ levels in culture supernatants were measured using ELISA kits from BD Biosciences, according to the manufacturer’s instructions.

### Infection protocol, parasitemia, and survival

For infection, different mouse hosts, parasite strains, and route of infection were assessed. BALB/c mice were challenged i.p. with 2000 or 3000 bloodstream trypomastigotes of the Tulahuen strain or with 20000 cultured trypomastigotes of the Dm28c strain, as detailed in the results, 15 days after the last TSf-ISPA immunization. C57BL/6 mice were i.p. infected with 1000 bloodstream trypomastigotes of the Tulahuen strain or with 20000 cultured trypomastigotes of the Dm28c strain, 15 days after the last TSf-ISPA immunization. Additionally, BALB/c and C57BL/6 mice were challenged with 2500 bloodstream trypomastigotes of the Tulahuen strain via i.d. injection in the footpad 15 days post-TSf-ISPA immunization.

Parasitemia was monitored at day 20 or 21 post-infection (p.i.) by examining 5 µl of blood directly under a microscope (Leica Microsystems), as previously described (26). Survival was recorded until day 21 post-infection in the case of flow cytometry studies or until day 35 p.i. according to the experiment.

### Statistical analysis

Normality was assessed using the Shapiro-Wilk test, and homogeneity of variances was evaluated with the Levene test. For comparisons between two groups, a t-test was applied when normality was not rejected, while ANOVA was used for comparisons involving more than two groups. In cases where ANOVA results were significant, Fisher’s test was employed to assess differences between columns. When normality was rejected, the Mann-Whitney test was used for two-group comparisons, and the Kruskal-Wallis test for multiple groups, with Dunn’s test for *post-hoc* comparisons. Depending on prior knowledge of how the variable was expected to change, one- or two-tailed tests were used in both the t-test and Mann-Whitney test. Survival curves were evaluated using the Mantel-Cox (Log-rank) test. Except for the Levene tests, which were performed using Python with the Pandas and SciPy libraries, all analyses were conducted using GraphPad Prism (GraphPad, California, USA). Statistical significance was indicated by an asterisk (*) for p < 0.05 between the specified groups. **Supplementary Table 2** provides the mean ± SD, exact p-values, and the tests used in each case.

## Results

We have previously shown that immunization with TSf adjuvanted with ISPA generated protection against *T. cruzi* infection, whereas TSf immunization alone or ISPA immunization alone did not improve parasitemia or survival compared to PBS-treated control mice (26). Interestingly, increases in CD11b+ Gr-1+ cells resembling MDSCs were observed at day 7 post TSf-ISPA immunization, suggesting that, despite TSf-ISPA eliciting significant protection, these cells could be interfering with the attainment of an even better protection of the vaccine candidate, as has also been suggested in several vaccine studies against other pathogens (27, 51–53).

### Suppressive capacity of MDSCs induced by TSf-ISPA immunization

To confirm that the Gr-1+ splenocytes that increase during the immunization phase exert suppressive capacity, *in vitro* assays were performed. Gr-1+ splenocytes from TSf-ISPA-immunized mice were purified by magnetic cell sorting and cultured with immature bone marrow-derived dendritic cells (BMDCs) alone or in the additional presence of splenocytes stained with CFSE. LPS was added to favor the maturation of BMDCs (50), and the suppressive capacity of the potential cells resembling MDSCs (as has been previously reported) (54). Additionally, LPS + ConA were used to analyze the suppressive capacity of purified Gr-1+ cells over CD4+ lymphocyte proliferation.

**Figure 1:**
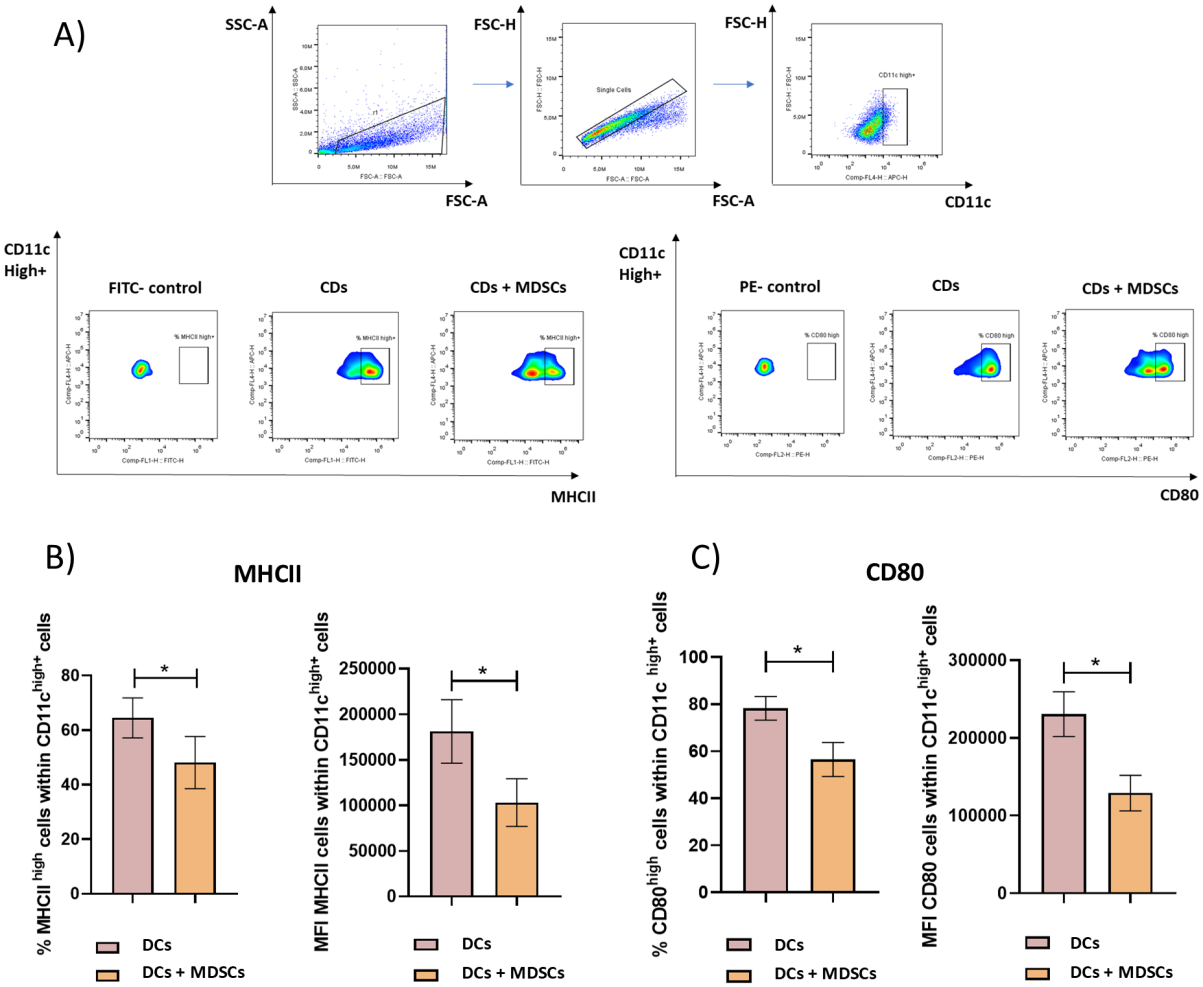
MDSC’s suppressive capacity over BMDCs. Gr-1+ splenocytes were purified by magnetic cell sorting from TSf-ISPA immunized mice, seven days after the last immunization dose. BMDCs were generated *in vitro* using standard protocols. Gr-1+ cells and BMDCs were cocultured in the presence of LPS (1µg/ml) for 3 days at 37°C and 5% CO_2_, and then were recovered and stained for flow cytometry. **(A)** Representative dot plots showing the gating strategy used to select CD11chigh+ cells and then analyze the suppressive capacity of Gr-1+ cells, assessing the expression of costimulatory molecules such as MHCII and CD80 within CD11chigh+ cells in the cocultured with Gr+1 cells (DCs + MDSCs) or not cocultured with Gr-1+ cells (DCs). **(B)** Percentage of MHCII^high^ cells within CD11chigh+ cells and MFI of MHCII within CD11chigh+ cells in the wells cocultured with Gr+1 cells (DCs + MDSCs) or not cocultured with Gr-1+ cells (DCs) (Data are expressed as means + standard deviations). **(C)** Percentage of CD80high cells within CD11chigh+ cells and MFI of CD80 within CD11chigh+ cells in the wells cocultured with Gr+1 cells (DCs + MDSCs) or not cocultured with Gr-1+ cells (DCs) (Data are expressed as means ± standard deviations) (n=3-4 wells per group). The result is representative of two independent experiments. *p< 0.0288, t-test for **(B)** left, *p<0.0286, Mann-Whitney test for **(B)** right, *p< 0.0288, t-test for **(C)**, in both cases.

**Figure 1A** shows the gating strategy used to analyze the expression of MHCII and CD80 by CD11chigh+ BMDCs in the presence or absence of purified Gr-1+ cells. A marked decrease in the percentage of MHCII^high^ and CD80^high^ cells within CD11chigh+ cells was observed when those cells were cocultured with Gr-1+ purified cells from immunized mice (**Figures 1B, C**). In the same line, a clear and significant decrease in the mean fluorescence intensity (MFI) of MHCII and CD80 within CD11chigh+ cells were observed when those cells were cocultured with Gr-1+ purified cells (**Figures 1B, C**).

**Figure 2:**
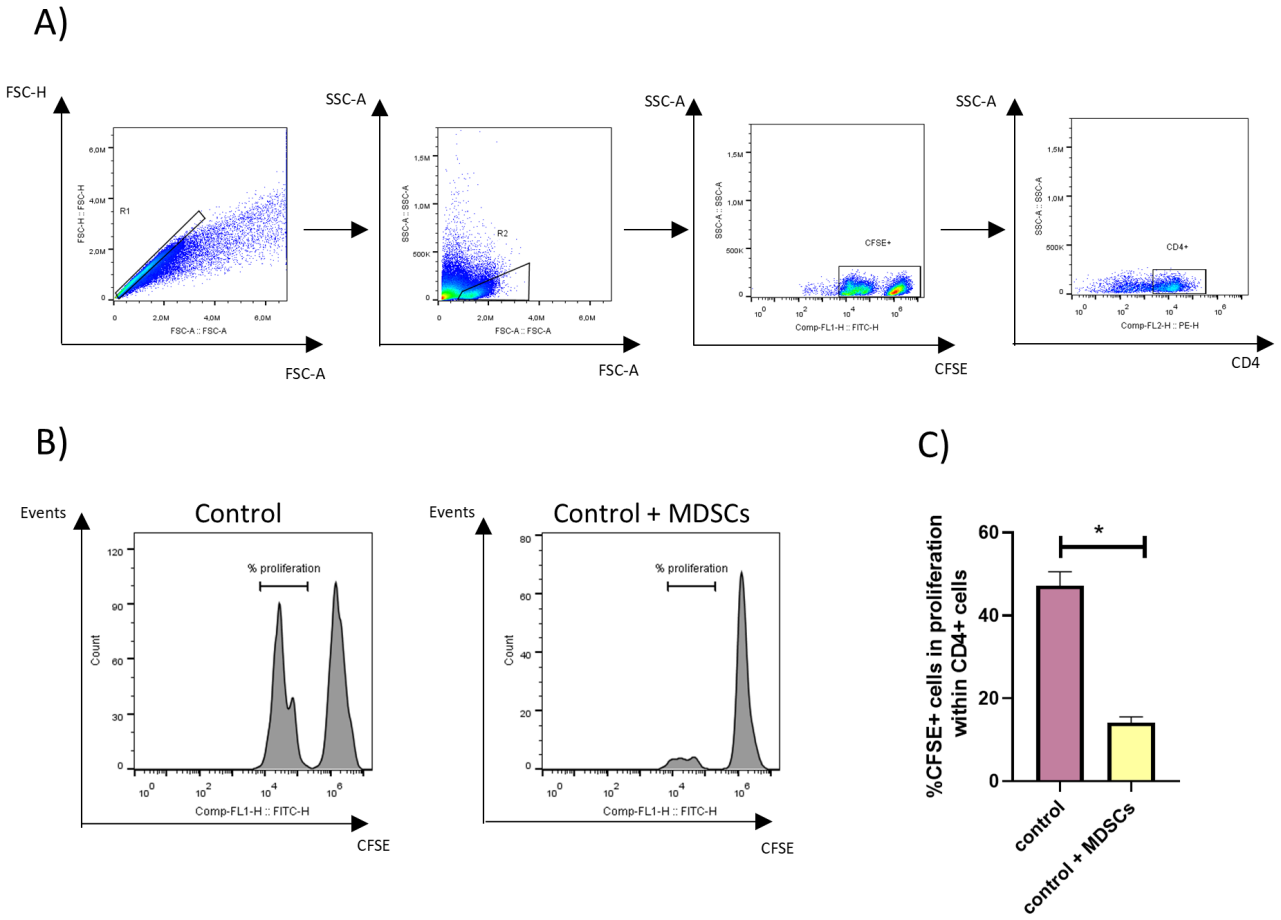
MDSC’s suppressive capacity over CD4+ lymphocytes. Gr-1+ splenocytes were purified by magnetic cell sorting from TSf-ISPA immunized mice, seven days after the last immunization dose. BMDCs were generated *in vitro* using standard protocols, and splenocytes from untreated mice were stained with CFSE. Gr-1+ cells, BMDCs, and splenocytes from untreated mice were cocultured in the presence of LPS and ConA for 3 days at 37°C and 5% CO_2_, and then were recovered and stained for flow cytometry. **(A)** Representative dot plots showing the gating strategy used to select CD4+ CFSE+ cells. **(B)** Representative histograms analyzing the suppressive capacity of MDSCs, assessing the proliferation of CFSE+ cells within CD4+ cells cocultured with Gr-1+ cells (Control + MDSCs) or not cocultured with Gr-1+ cells (control). **(C)** Percentage of proliferating CFSE+ cells within CD4+ cocultured with Gr-1+ cells (control + MDSCs) or not cocultured with Gr-1+ cells (Control) (Data are expressed as means ± standard deviations) (n=3 wells per group) *p<0.001, t-test. The result is representative of two independent experiments.

Additionally, CFSE-labeled splenocytes were incorporated into the wells containing BMDCs and Gr-1+ purified cells to analyze the proliferation of CD4+ lymphocytes. **Figure 2A** shows the gating strategy employed, **Figure 2B** displays representative histograms, and **Figure 2C** displays the percentage of proliferating CFSE+ cells within CD4+ splenocytes. Results obtained showed that the proliferation of ConA-stimulated CD4+ lymphocytes was suppressed in the presence of Gr-1+ purified cells from TSf-ISPA-immunized mice.

Taken together, these results provide evidence supporting that the Gr-1+ cells that increase during the TSf-ISPA immunization phase include MDSCs with suppressive capacity.

### Kinetics of MDSCs in TSf-ISPA immunization protocols with and without 5FU

Although several studies have shown that immunization can elicit MDSCs with the ability to limit the efficacy of vaccine candidates (27), to our knowledge, the analysis of the kinetics of MDSCs has not been addressed yet. Thus, considering that a better understanding of the alterations of MDSCs at this stage may favor rational vaccine design, a deeper study of the MDSC dynamics during TSf-ISPA immunization was conducted.

**Figure 3:**
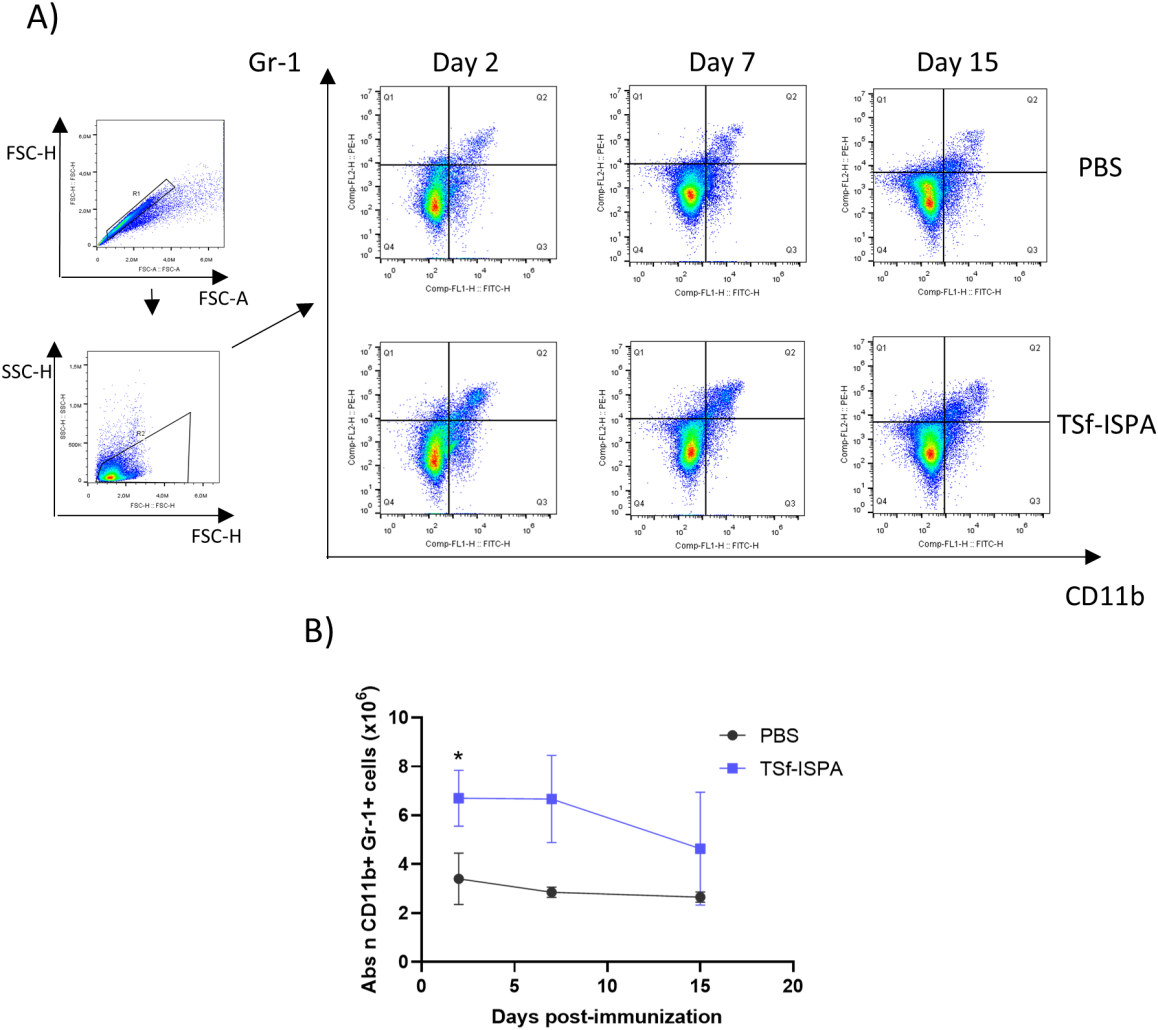
Alterations in the CD11b+ Gr-1+ splenocytes at different days post-immunization of TSf-ISPA mice. Mice were treated with TSf-ISPA or PBS three times every two weeks and were sacrificed to obtain splenocytes at different days after the last immunization dose. **(A)** Dot plots showing the selected regions for the analysis of Gr-1+ and CD11b+ cells among groups at different days post-imm. **(B)** Kinetics of the absolute number (abs n) of CD11b+ Gr-1+ cells at different days post-imm. (2, 7, and 15 days) in splenocytes from mice treated with PBS control solution or TSf-ISPA (data are expressed as means ± standard deviations). (n=3-5 mice per group). *p=0.0015, TSf-ISPA vs PBS at day 2 post-imm, t-test.

**Figure 3A** shows representative dot plots of the expression of CD11b and Gr-1 markers in splenocytes at 2, 7, and 15 days post-imm. with TSf-ISPA. As shown in **Figure 3B**, the absolute number of CD11b+ Gr-1+ splenocytes as well as the percentage of those cells (**Supplementary Figure 2A**) were early increased at day 2 after the last immunization dose, then remained increased at day 7 post-imm., and finally decreased by day 15 post-imm. This result suggests that TSf-ISPA immunization generate a marked increase of MDSCs that decrease by day 15 post-imm.

In addition, we have previously reported that i.p. pretreatment with 5FU before TSf-ISPA immunization increased the protective capacity of the formulation (25). The selective effect of the dose of 5FU employed to target MDSCs has been reported previously, and even in the context of *T. cruzi* infection (21, 22, 29, 48).

**Figure 4:**
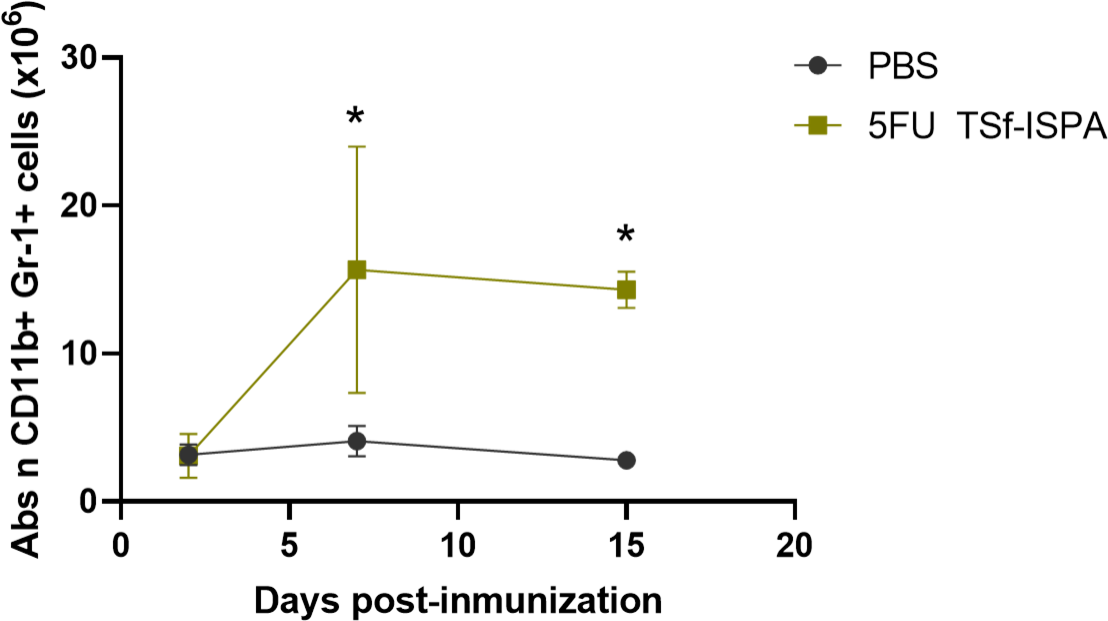
Alterations of CD11b+ Gr-1+ splenocytes at different days post-immunization with 5FU TSf-ISPA. Mice were treated with 5FU TSf-ISPA or PBS three times every two weeks and were sacrificed to obtain splenocytes at different days after the last immunization dose. Kinetics of the absolute number (Abs n) of CD11b+Gr-1+ cells at different days post-imm. (2, 7, and 15 days) in splenocytes from mice treated with PBS or 5FU TSf-ISPA. (data are expressed as means ± standard deviations) (n=3-5 mice per group). *p=0.0149 5FU TSf-ISPA vs PBS group day 7, t-test; *p<0.001, 5FU TSf-ISPA vs PBS group day 15, t-test.

In this study, we seek to elucidate how 5FU administration modifies the kinetics of CD11b+ Gr-1+ MDSCs during TSf-ISPA immunization. **Figure 4B**, **Supplementary Figure 2B** shows that 5FU treatment was clearly effective, avoiding an early increase of CD11b+ Gr-1+ cells at day 2 post TSf-ISPA immunization. However, it was noteworthy that this effect was transitory and both the absolute number (**Figure 4B**) and percentage (**Supplementary Figure 2B**) of CD11b+ Gr-1+ splenocytes increased markedly by day 7 post-imm., likely as a rebound effect, as has also been observed in other infection processes (55), a fact that could be associated with a strong immune effector response elicited in the early absence of MDSCs. Then, at day 15 post-imm., the level of CD11b+ Gr-1+ cells slightly decreased, yet it remained significantly higher compared to the levels observed in control mice (**Figure 4**, **Supplementary Figure 2B**).

**Figure 5:**
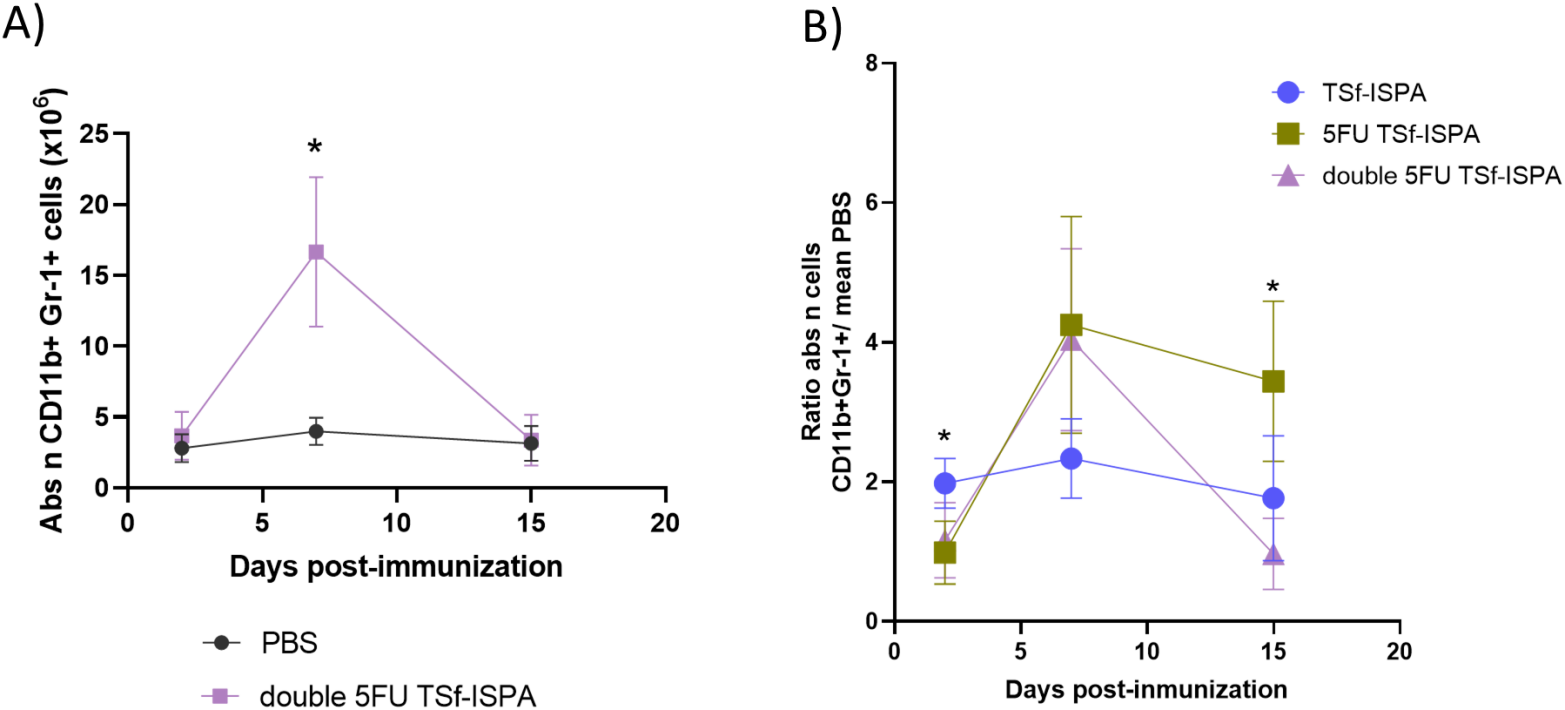
Alterations of CD11b+ Gr-1+ splenocytes at different days post-immunization with double 5FU TSf-ISPA and ratios against PBS-treated mice. Mice were treated with double 5FU TSf-ISPA or PBS three times every two weeks and were sacrificed to obtain splenocytes at different days after the last immunization dose. **(A)** Kinetics of the absolute number (abs n) of CD11b+Gr-1+ cells at different days post-imm. (2, 7, and 15 days) in splenocytes from mice treated with PBS or double 5FU TSf-ISPA. (data are expressed as means ± standard deviations) (n=5). *p< 0.01, double 5FU TSf-ISPA vs PBS, day 7, t-test. **(B)** Ratio of the absolute number (abs n) of CD11b+ Gr-1+ cells from each mouse from a group (TSf-ISPA or 5FU TSf-ISPA or double 5FU TSf-ISPA) divided by the mean of the percentage in the PBS group (data are expressed as means ± standard deviations) (n=3-5 mice per group). ANOVA at day 2 post-imm: p=0.0081. Comparison between groups: *p= 0.0032, Fisher’s test between TSf-ISPA vs 5FU TSf-ISPA; and *p=0.013, Fisher’s test between TSf-ISPA vs double 5FU TSf-ISPA. ANOVA at day 15 post-imm: *p=0.0043. *p=0.0027 between TSf-ISPA vs 5FU-TSf-ISPA, Fisher’s test, and *p= 0.0014 5FU TSf-ISPA vs double 5FU TSf-ISPA, Fisher’s test.

The knowledge obtained regarding the kinetics of MDSCs after 5FU treatment opened the possibility to rationally design new protocols to improve the TSf-ISPA vaccine candidate. Thus, to attempt to tackle the increase of MDSCs observed at day 7 post-imm., a second dose of 5FU was administered at day 7 post-imm., in a protocol termed double 5FU TSf-ISPA. As shown in **Figure 5A**, **Supplementary Figure 2C**, the absolute number and percentage of CD11b+ Gr+1+ splenocytes were close to basal levels at day 2 post-imm. Subsequently, there was a significant increase at day 7 post-imm. However, in this case, because of the second dose of 5FU, CD11b+ Gr-1+ cells finally decreased to almost basal levels at day 15 post-imm (**Figure 5A**, **Supplementary Figure 2C**).

To compare all the protocols simultaneously, a ratio was calculated by dividing the absolute number and percentage of MDSCs in each mouse from every group by the mean values of the control group treated with PBS.

It is interesting to note that at day 2 post-imm. both groups of mice treated with 5FU before TSf-ISPA immunization decreased the absolute number (**Figure 5B**) and percentage (**Supplementary Figure 2D**) of MDSCs compared to TSf-ISPA vaccinated mice. Then, at day 7 post-imm., all groups of treated mice generated an increase of MDSCs compared to control mice, with a ratio similar or higher than 2 regarding the absolute number and percentage of MDSCs (**Figure 5B**, **Supplementary Figure 2D**). Finally, the second dose of 5FU at day 7 post-imm. caused a marked modification in the dynamics of MDSCs in double 5FU TSf-ISPA versus 5FU TSf-ISPA mice, which received only one dose of the drug. In this sense, a significant decrease in the percentage and absolute number of CD11b+ Gr-1+ splenocytes in double 5FU TSf-ISPA mice was observed compared to 5FU TSf-ISPA mice, strongly suggesting that the second dose is necessary to effectively decrease the expansion of those cells (**Figure 5**, **Supplementary Figure 2D**). Both subtypes of MDSCs, CD11b+Ly6G+Ly6C+/low (G-MDSCs) and CD11b+Ly6G-Ly6C+ (M-MDSCs) were increased at day 7 post-imm in 5 FU TSf-ISPA and double 5FU TSf-ISPA vaccinated mice (**Supplementary Figure 3**), in line with the increases registered measuring CD11b and Gr-1 markers.

### Comparison of protective efficacy in TSf-ISPA immunization with one or two doses of 5FU for optimal protocol selection

**Figure 6:**
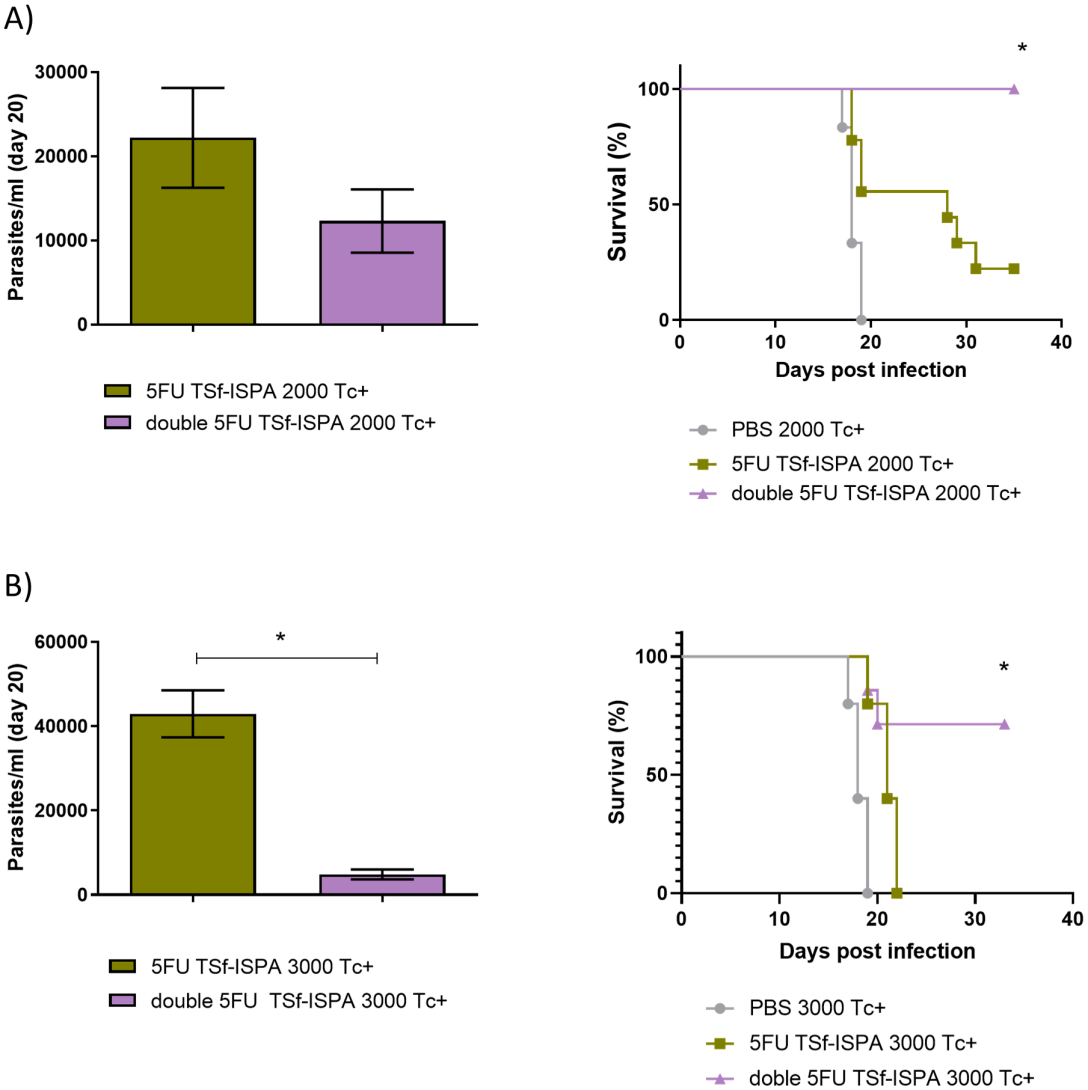
Evaluation of parasitemia and survival of 5FU TSf-ISPA and double 5FU TSf-ISPA BALB/c mice infected with 2000 and 3000 trypomastigotes from the Tulahuen strain. **(A)** Parasitemia and survival of 5FU TSf-ISPA and double 5FU TSf-ISPA immunized mice, which were infected with 2000 trypomastigotes of the Tulahuen strain of *T. cruzi*, 15 days after the last immunization dose. Parasitemia is shown at day 20 post-infection (Data are expressed as means ± standard deviations), all PBS mice died before day 20 post-infection. Survival is shown until day 35 post-infection (n=5-7) *p<0.05 between double 5FU TSf-ISPA vs 5FU TSf-ISPA or PBS, Log rank-test. **(B)** Parasitemia and survival of 5FU TSf-ISPA and double 5FU TSf-ISPA immunized mice, which were infected with 3000 trypomastigotes of the Tulahuen strain of *T. cruzi*. Parasitemia is shown at day 20 post-infection (Data are expressed as means ± standard deviations), *p=0.0357, Mann-Whitney (all PBS mice died before day 20-post-infection). Survival is shown until day 35 post-infection (n=5-9) *p<0.05 between double 5FU TSf-ISPA vs 5FU TSf-ISPA or PBS, (logrank-test).

According to these results, a challenge with a very high dose of parasites was employed to assess whether the decrease of MDSCs obtained with 2 doses of 5FU was able to improve the protective capacity of the TSf-ISPA vaccine candidate. Since we have previously reported that mice vaccinated with 5FU TSf-ISPA survived 100% against an i.p. challenge with 1500 parasites (25), a high lethal dose of 2000 and 3000 parasites was used in this study. As shown in **Figure 6A**, when 2000 parasites were used, the survival of the 5FU TSf-ISPA group was nearly 20%, whereas double 5FU TSf-ISPA mice showed a higher protective capacity and still remained at 100% survival. In addition, a dose of 3000 parasites was strong enough to cause 100% mortality in 5FU-TSf-ISPA mice, whereas double 5FU TSf-ISPA showed 71% survival (**Figure 6B**). Additionally, both parasitemia studies also supported the results observed regarding survival, and double 5FU TSf-ISPA treated mice showed lower levels of parasitemia compared to mice that received the 5FU-TSf-ISPA vaccine. As expected, PBS-treated control mice were unable to withstand such a high dose of parasites and had 100% mortality even before day 20 of infection.

Taken together, these results strongly support that two doses of 5FU to deplete MDSCs during the immunization phase are able to markedly potentiate the TSf-ISPA vaccine candidate, even when high lethal doses of parasites are used.

### Comparison of the effector response during TSf-ISPA immunization with one or two doses of 5FU for optimal protocol selection

It has been reported that MDSC increases during immunization may limit the efficacy of vaccination, likely by suppressing dendritic cells and the elicitation of a more potent effector response (27, 54). To analyze whether the better protective capacity observed with 2 doses of 5FU was the consequence of a better effector immune response, several immune parameters were analyzed at day 7 post-imm. in both groups of mice treated with 5FU: 5FU TSf-ISPA mice and double 5FU TSf-ISPA.

**Figure 7:**
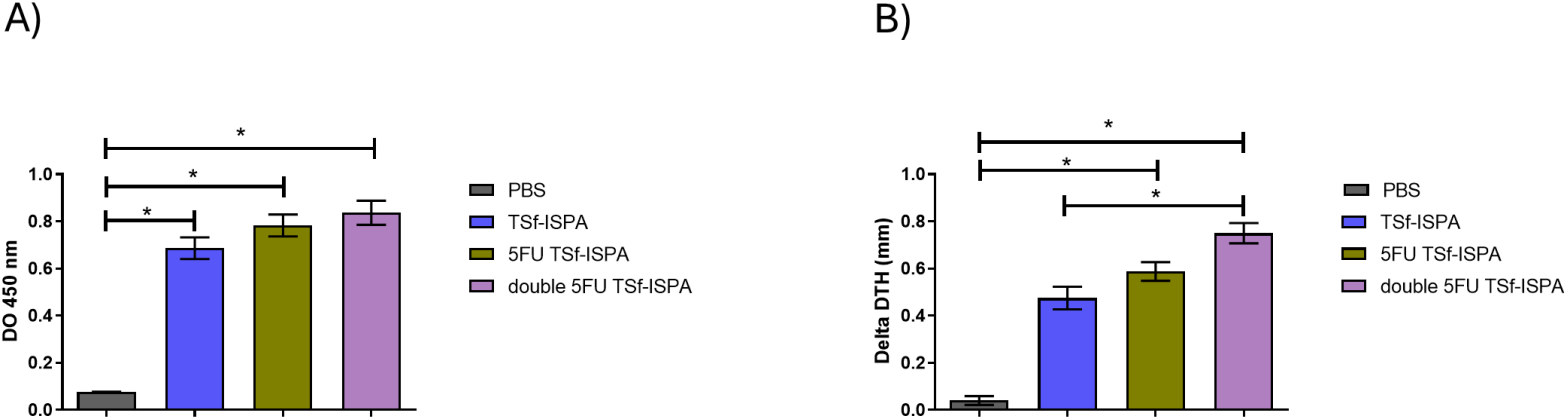
Immunological parameters of the immune response elicited by TSf-ISPA, 5FU TSf-ISPA and double 5FU TSf-ISPA immunization. **(A)**. BALB/c mice were immunized with PBS, TSf-ISPA, 5FU TSf-ISPA, or double 5FU TSf-ISPA, and plasma samples were analyzed for TSf-specific IgG antibodies by ELISA. ANOVA: *p<0.001, Fisher’s test: *p<0.001 between PBS and TSf-ISPA, or PBS vs 5FU TSf-ISPA, or PBS vs double 5FU TSf-ISPA. **(B)**. Delayed type hypersensitivity (DTH) response in immunized mice: Footpad thickness was measured before and 48 h after inoculation of 5 μg of TSf seven days after completion of the immunization schedule. Results are expressed as “delta DTH (mm),” which was the difference between the values obtained after and before inoculation. Data are expressed as means ± standard deviations. (n = 5-9 mice per group), Kruskal-Wallis *p=0.0001, *p=0.0071 Dunn´s test between PBS vs 5FU TSf-ISPA; *p <0.0001 Dunn´s t-test between PBS vs double 5FU TSf-ISPA; *p=0.001 Dunn´s test between TSf-ISPA vs double 5FU TSf-ISPA. Results shown are representative of 2 independent experiments.

Both groups of 5FU-vaccinated mice generated significant levels of anti-TSf IgG antibodies (**Figure 7A**) and specific cellular response (**Figure 7B**) compared with control PBS-treated mice. In addition, although no difference in antibody levels was observed at day 7 post-imm., double 5FU TSf-ISPA mice showed a significant increase in the DTH response compared to 5FU TSf-ISPA mice (**Figure 7B**).

**Figure 8:**
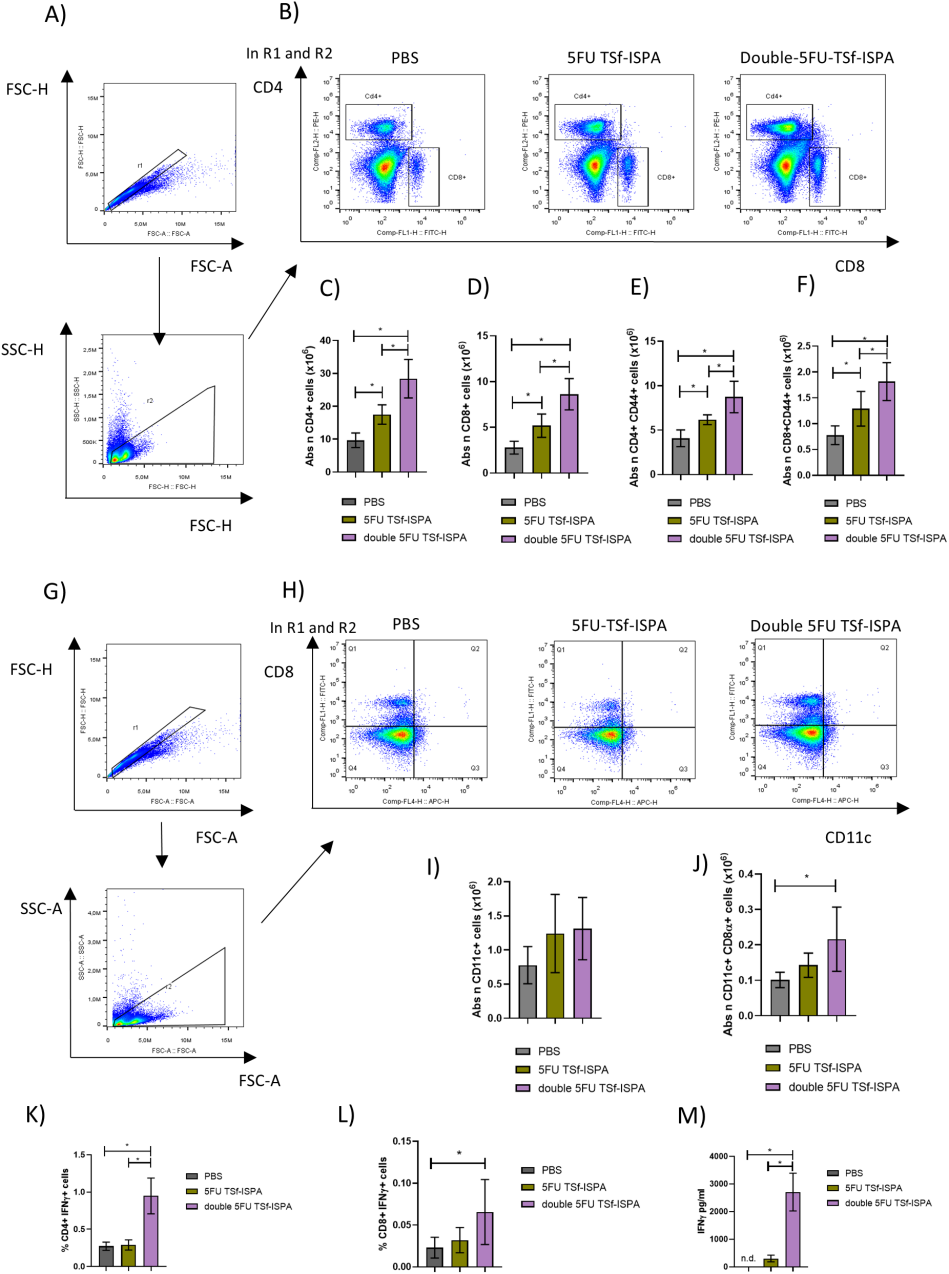
Changes in cell populations in spleens at 7 days post-immunization. Mice were treated with PBS, 5FU-TSf-ISPA, or double 5FU TSf-ISPA, and were sacrificed 7 days after the last immunization dose to obtain splenocyte suspensions. **(A, B)** Representative dot plots showing the strategy used to select the CD4+, CD8+ populations in experimental and control PBS-treated mice. **(C)** Absolute number of CD4+ splenocytes (x10^6^) ANOVA: *p<0.0001, *p=0,0091 between PBS vs 5FU TSf-ISPA; *p<0.0001 between PBS vs double 5FU TSf-ISPA; *p=0.001 between 5FU TSf-ISPA vs double 5FU TSf-ISPA, Fisher´s test. **(D)** Absolute number of CD8+ cells (x10^6^). ANOVA: *p<0.0001, *p=0.0126 between PBS vs 5FU TSf-ISPA; *p<0.0001 between PBS vs double 5FU TSf-ISPA; *p=0,0013 between 5FU TSf-ISPA vs double 5FU TSf-ISPA, Fisher´s test **(E)** Absolute number of CD4+CD44+ cells (x10^6^). ANOVA: *p=0.0002, *p=0.0175 between PBS vs 5FU TSf-ISPA; *p<0.0001 between PBS vs double 5FU TSf-ISPA; *p=0,0055 between 5FU TSf-ISPA vs double 5FU TSf-ISPA, Fisher´s test **(F)** Absolute number of CD8+CD44+ cells (x10^6^). ANOVA: *p=0.0006, *p=0.0213 between PBS vs 5FU TSf-ISPA; *p=0.0002 between PBS vs double 5FU TSf-ISPA; *p=0,0186 between 5FU TSf-ISPA vs double 5FU TSf-ISPA, Fisher´s test. **(G, H)** Representative dot plots showing the strategy used to select the CD11c+ and CD11c+ CD8α+ populations in experimental and control PBS-treated mice, **(I)** Absolute number of CD11c+ cells (x10^6^). **(J)** Absolute number of CD11c+CD8α+ cells (x10^6^), ANOVA: *p=0.0245, *p=0.082 between PBS vs double 5FU TSf-ISPA, Fisher´s test. Data are expressed as means ± standard deviations. Results shown are representative of 2 independent experiments (n =3-5 mice per group). **(K-M)** *In vitro* culture of splenocytes. After 72 hours of incubation with TSf, CD4+IFN-γ+ cells, CD8+IFN-γ+ cells, and IFN-γ levels in supernatants were measured. **(K)** Percentage of CD4+IFN-γ+ cells. ANOVA *p < 0.001, *p < 0.0001 between PBS vs. double 5FU TSf-ISPA; *p < 0.0001 between 5FU TSf-ISPA vs. double 5FU TSf-ISPA (Fisher’s test). **(L)** Percentage of CD8+IFN-γ+ cells. ANOVA *p = 0.083, *p = 0.039 between PBS vs. double 5FU TSf-ISPA (Fisher’s test). **(M)** IFN-γ levels in supernatants (pg/mL). ANOVA *p = 0.0003, p < 0.0002 between PBS vs. double 5FU TSf-ISPA; p = 0.0003 between 5FU TSf-ISPA vs. double 5FU TSf-ISPA (Fisher’s test).

Vaccinated mice were sacrificed at day 7 after the last immunization dose to analyze quantitative changes in several splenic immune populations. **Figures 8A, B** shows the gating strategy used to analyze the changes in CD4+, CD8+, CD11c+ CD11c+CD8α+ cells (**Supplementary Figure 4** shows the gating strategy used to analyze CD4+CD44+ and CD8+CD44+ cells). Interestingly, double 5FU-treated mice elicited the highest increase in the absolute number of CD4+, CD4+CD44+, CD8+, CD8+CD44+ cells (**Figures 8C–F**), and a trend supporting the elicitation of a higher increase of CD11c+ and CD11c+CD8α+ splenocytes (**Figures 8G–J**). (Percentages of all analyzed populations are shown in **Supplementary Figure 4**). In line with these results, cultured splenocytes from mice immunized with double 5FU TSf-ISPA showed a higher percentage of CD4+IFN-γ+ cells and a trend toward a similar increase in CD8+IFN-γ+ cells after culture with TSf, compared to splenocytes from 5FU TSf-ISPA mice (**Figures 8K, L**). Additionally, culture supernatants from double 5FU TSf-ISPA mice exhibited higher levels of IFN-γ than those from 5FU TSf-ISPA mice, as measured by ELISA (**Figure 8M**).

**Figure 9:**
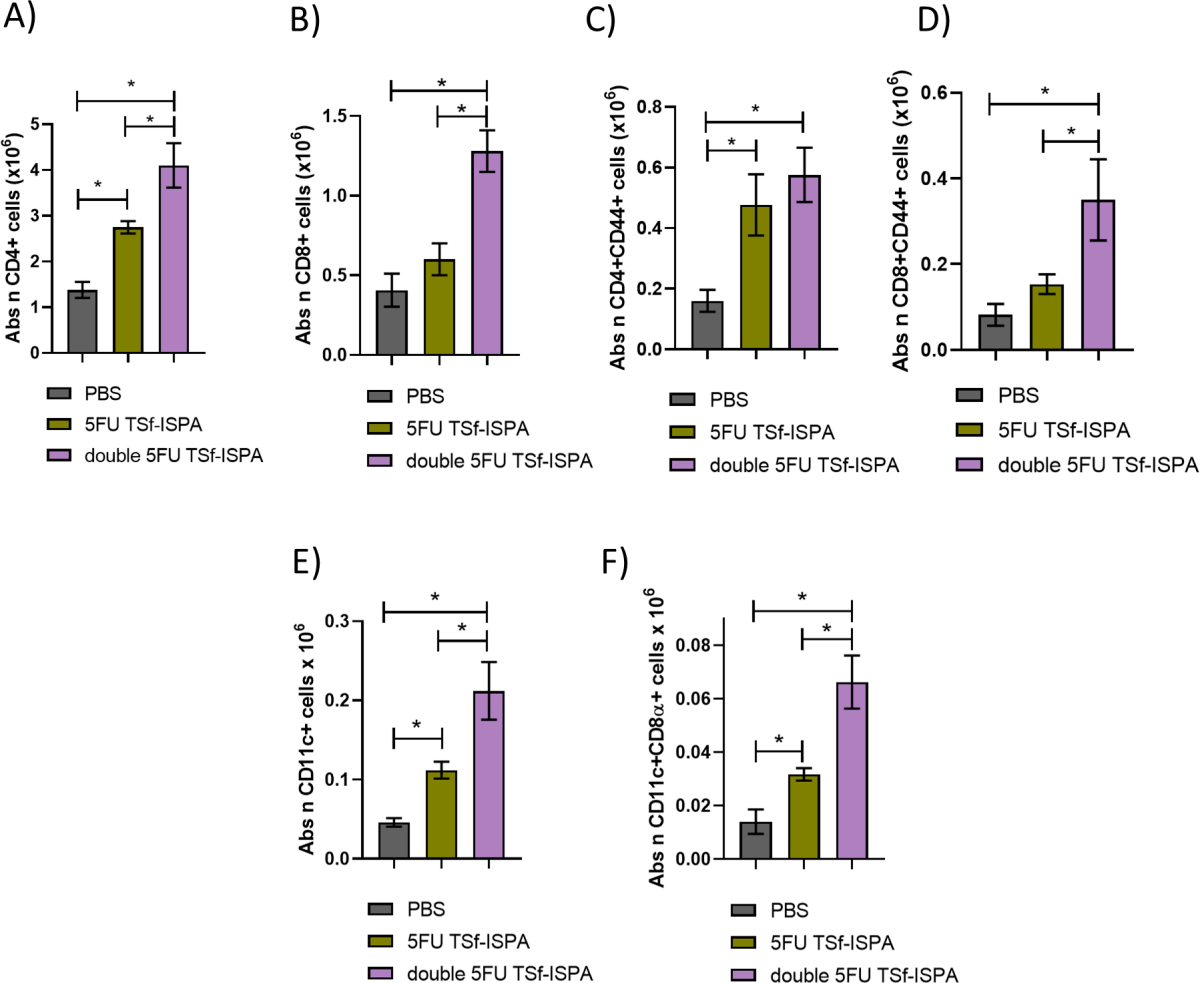
Changes in cell populations in lymph nodes at 7 days post-immunization. Mice were treated with PBS, 5FU-TSf-ISPA, or double 5FU TSf-ISPA, and were sacrificed 7 days after the last immunization dose to obtain lymph node cell suspensions. **(A)** Absolute number of CD4+ lymph node cells (x10^6^), ANOVA: *p<0.0001, *p=0.0016 between PBS vs 5FU TSf-ISPA; *p<0.0001 between PBS vs double 5FU TSf-ISPA; *p=0.0009 between 5FU TSf-ISPA vs double 5FU TSf-ISPA, Fisher´s test. **(B)** Absolute number of CD8+ cells (x10^6^), *ANOVA: p<0.0001 between PBS vs double 5FU TSf-ISPA; *p<0.0001 between 5FU TSf-ISPA vs double 5FU TSf-ISPA, Fisher´s test. **(C)** Absolute number of CD4+ CD44+ cells (x10^6^). ANOVA: *p<0.0004, *p=0.0016 between PBS vs 5FU TSf-ISPA; *p=0.0001 between PBS vs double 5FU TSf-ISPA, Fisher´s test **(D)** Absolute number of CD8+ CD44+ cells (x10^6^) ANOVA: *p=0.0015; *p<0.0007 between PBS vs double 5FU TSf-ISPA; *p=0.0046 between 5FU TSf-ISPA vs double 5FU TSf-ISPA, Fisher´s test. **(E)** Absolute number of CD11c+ cells (x10^6^). ANOVA: *p<0.0001, *p=0.0154 between PBS vs 5FU TSf-ISPA; *p<0.0001 between PBS vs double 5FU TSf-ISPA; *p=0.0008 between 5FU TSf-ISPA vs double 5FU TSf-ISPA, Fisher´s test **(F)** Absolute number of CD11c+ CD8α+ cells (x10^6^). ANOVA: *p<0.0001, *p=0.0201 between PBS vs 5FU TSf-ISPA; *p<0.0001 between PBS vs double 5FU TSf-ISPA; *p=0.0002 between 5FU TSf-ISPA vs double 5FU TSf-ISPA, Fisher´s test. Data are expressed as means ± standard deviations. Results shown are representative of 2 independent experiments (n =3-5 mice per group).

Supporting the analysis performed in the spleen, very similar results were observed when the absolute number of lymph node cells from 5FU TSf-ISPA versus double 5FU TSf-ISPA vaccinated mice was analyzed. In all cases, double 5FU TSf-ISPA mice showed higher numbers of CD4+, CD4+CD44+, CD8+, CD8+CD44+, CD11c+, and CD11c+CD8α+ (**Figures 9A–F**), also supporting that double administration of 5FU potentiates the effector response caused by the TSf-ISPA vaccine.

Taken together, these results support the notion that the immunization protocol based on two doses of 5FU not only decreased MDSCs during immunization but also generated an increase in the absolute number of several effector immune populations in the spleen and lymph nodes of vaccinated mice, correlating with the higher protection observed when the double 5FU TSf-ISPA protocol was compared to 5FU TSf-ISPA, based on one dose of 5FU.

### Study of MDSCs after infection of 5FU-treated TSf-ISPA immunized mice

**Figure 10:**
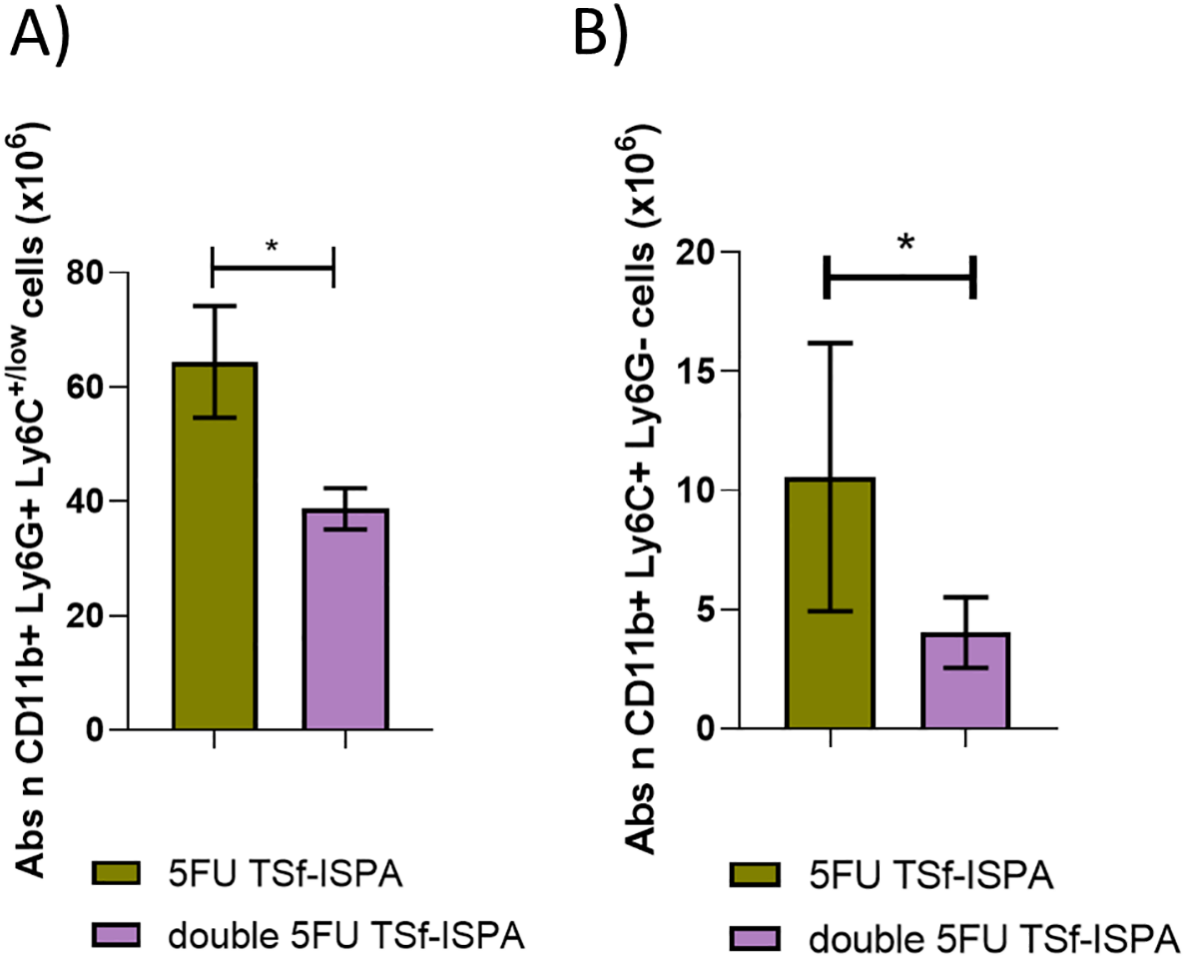
Changes in spleen G-MDSCs and M-MDSCs at day 21 post *T. cruzi* infection. Expression of CD11b+ Ly6G+ and Ly6C+ was analyzed by flow cytometry in 5FU TSf-ISPA immunized and double 5FU TSf-ISPA immunized mice. **(A)** Absolute number of CD11b+ Ly6G+ Ly6C+/low (G-MDSCs) cells (x10^6^). *p= 0.0163 t-test. **(B)** Absolute number of CD11b+ Ly6G- Ly6C+ (M-MDSCs) cells (x10^6^) *p= 0.0286, Mann-Whitney test. Data are expressed as means ± standard deviations (n = 4 per group).

Moreover, we have previously shown that TSf-ISPA vaccinated mice (without 5FU) already have higher survival than control mice, correlating with lower levels of MDSCs after *T. cruzi* infection (26). To also analyze whether the protective effect of two doses of 5FU during immunization was evidenced by a decrease in MDSCs after infection, an additional assay was performed. In addition, and similarly to our previous study, both subtypes of MDSCs were measured G-MDSCs (CD11b+, Ly6G+ Ly6C+/low), and M-MDSCs, (CD11b+ Ly6G- Ly6C+). In accordance with all the results described herein, double 5FU TSf-ISPA vaccinated and infected mice showed a lower absolute number of G-MDSCs and M-MDSCs splenocytes as compared to 5FU TSf-ISPA vaccinated and infected mice (**Figure 10**). **Supplementary Figures 5A, B** show the percentages of G-MDSCs and M-MDSCs. Thus, taken together, these results support that rational control of MDSC dynamics during immunization caused an increased effector response, and then, after infection, TSf-ISPA vaccinated mice that received a double dose of 5FU showed lower levels of MDSCs correlating with lower parasitemia and better survival after *T. cruzi* infection.

### Protective capacity of the double 5FU TSf-ISPA immunization in multiple murine models

To date, very few studies have assessed the protective capacity of vaccine candidates against *T. cruzi* from different DTUs. Given the genetic diversity of the parasite, this may constitute an important aspect to be considered when evaluating the usefulness of a vaccine candidate. Taking into consideration the high level of protection obtained by the double 5FU TSf-ISPA vaccine in the model of BALB/c mice i.p. infected with the Tulahuen *T. cruzi* strain, the protective capacity of the formulation was also assessed in several different preclinical models.

**Figure 11:**
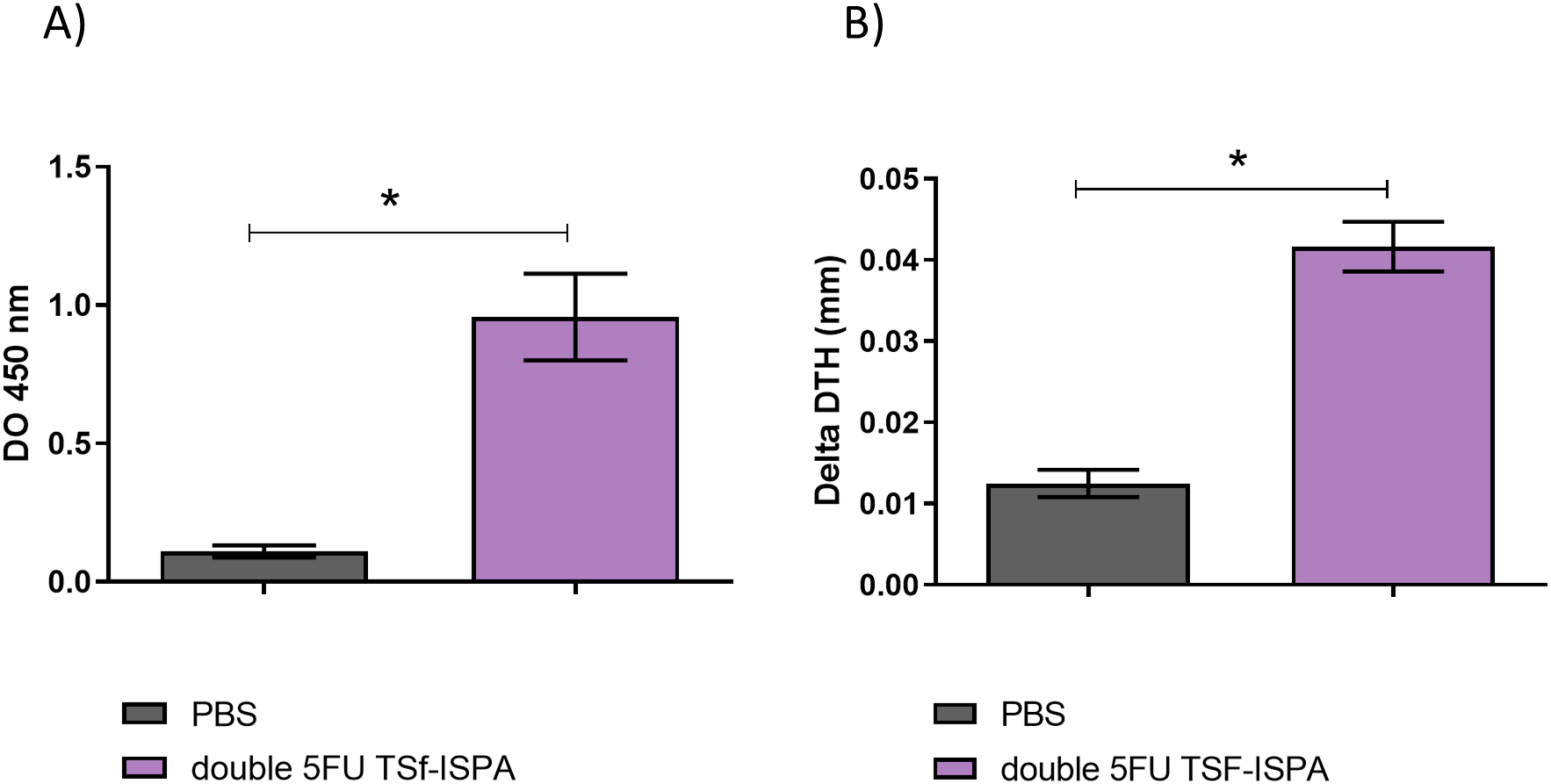
Immunological parameters of the immune response elicited by double 5FU TSf-ISPA immunization of C57BL/6 mice. **(A)** C57BL/6 mice were inoculated with PBS or double 5FU TSf-ISPA, and plasma samples were analyzed for TSf-specific IgG antibodies by ELISA. *p=0.0022, Mann-Whitney. **(B)** DTH response in immunized mice: Footpad thickness was measured before and 48 h after inoculation of 5 μg of TSf seven days after completion of the immunization schedule. Results are expressed as “delta DTH (mm),” which was the difference between the values obtained after and before inoculation *p= 0.0011, Mann-Whitney. Data are expressed as means ± standard deviations. Results shown are representative of 2 independent experiments (n = 5 mice per group).

To initiate this type of analysis, we first assessed the immune response generated by the double 5FU TSf-ISPA vaccine in C57BL/6 mice and then evaluated the protective capacity of the formulation against i.p. Tulahuen *T. cruzi* infection. The vaccine protocol was able to elicit significant levels of anti-TSf IgG antibodies (**Figure 11A**) and a strong DTH response (**Figure 11B**), in line with the results obtained in the model of BALB/c mice immunization. Thus, the results described herein support the capacity of the vaccine protocol to elicit immune responses in both strains of mice, BALB/c and C57BL/6.

**Figure 12:**
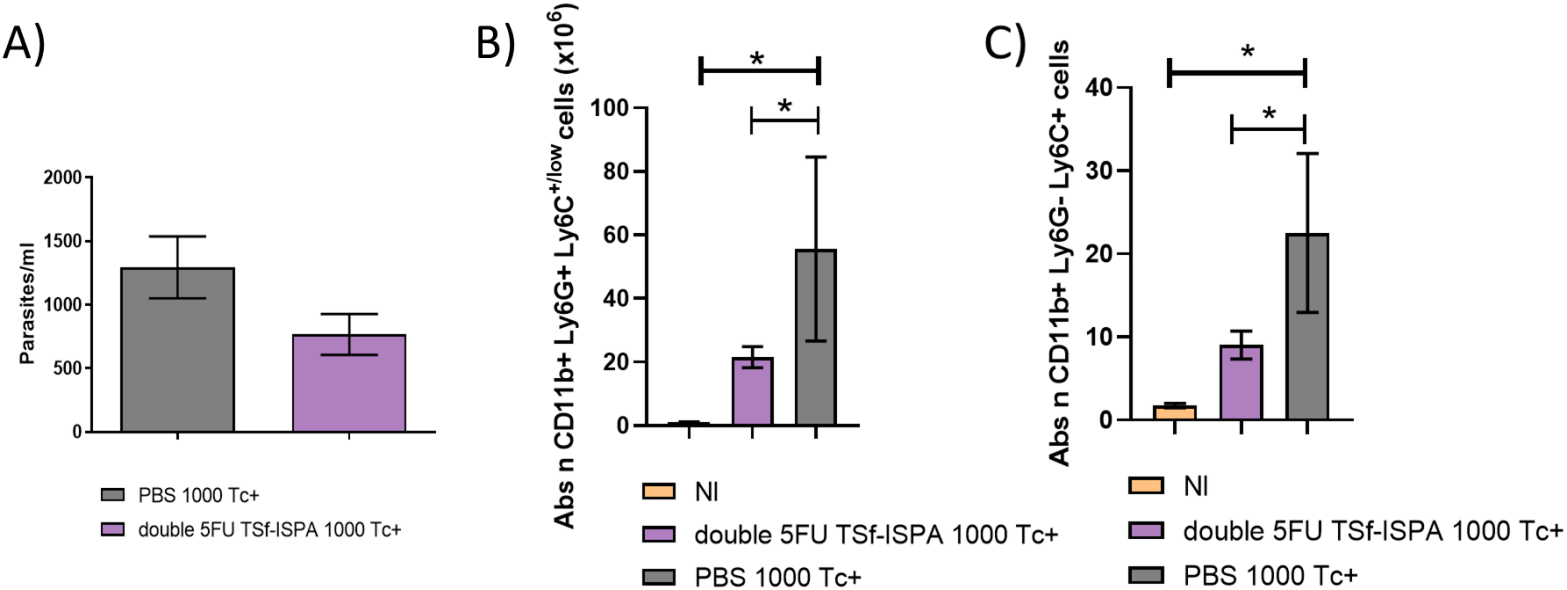
Changes in parasitemia and spleen G-MDSCs and M-MDSCs at day 21 post *T. cruzi* infection of C57BL/6 mice. **(A)** Parasitemia of PBS and double 5FU TSf-ISPA immunized C57BL/6 mice, which were infected (Tc+) with 1000 trypomastigotes of the Tulahuen strain of *T. cruzi* (DTU VI), 15 days after the last immunization dose. Parasitemia is shown at day 20 post-infection (data are expressed as means + standard deviations). Expression of CD11b, Ly6G and Ly6C was analyzed by flow cytometry in non-immunized and non-infected mice (NI), PBS-treated, and double 5FU TSf-ISPA immunized mice. **(B)** Absolute number of CD11b+ Ly6G+ Ly6C+/low (G-MDSCs) cells (x10^6^), ANOVA: *p<0.0001, *p=0.0009 NI vs PBS, *p= 0.0201 double 5FU TSf-ISPA vs PBS, Fisher´s test, **(C)** absolute number of CD11b+ Ly6G- Ly6C+ (M-MDSCs) cells (x10^6^) ANOVA: *p<0.001, *p= 0.0005 NI vs PBS, *p= 0.029 double 5FU TSf-ISPA vs PBS, Fisher´s test. Data are expressed as means ± standard deviations (n =3-5 per group).

C57BL/6 mice were then challenged with 1000 *T. cruzi* of Tulahuen strain (DTU VI) via the i.p. route. This dose did not cause mortality in control PBS-treated mice nor in vaccinated mice. Regarding parasitemia, there was a trend supporting that double 5FU TSf-ISPA vaccinated mice developed a lower level of parasitemia (**Figure 12A**). To analyze whether better protection with lower parasitemia correlated with lower levels of MDSCs after infection, mice were sacrificed at day 21 post-infection. A significant decrease in the number of both subtypes of MDSCs (G-MDSCs and M-MDSCs) was observed in double 5FU TSf-ISPA vaccinated mice, as compared to PBS-treated and infected mice (**Figures 12B, C**). **Supplementary Figure 6** displays that the percentage of MDSCs was also lower in vaccinated mice. Therefore, once again, these results are also in accordance with our previously published results and other results described herein, in which vaccinated mice were protected against *T. cruzi* correlating with lower levels of spleen MDSCs (25, 26).

**Figure 13:**
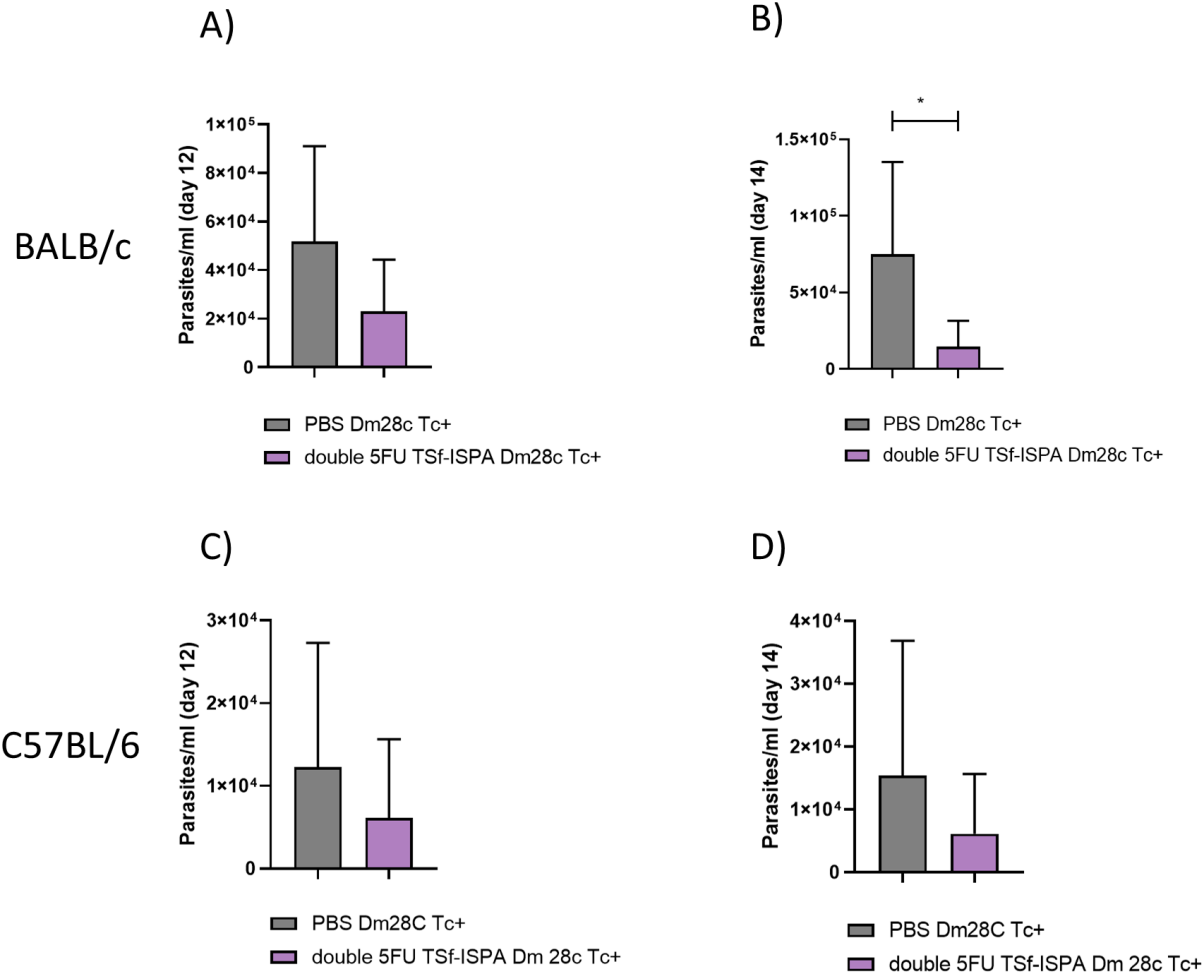
Changes in parasitemia at days 12 and 14 post Dm28c *T. cruzi* infection of BALB/c and C57BL/6 mice. **(A, B)** After Dm28c *T. cruzi* infection of BALB/c mice treated with PBS or double 5FU TSf-ISPA mice, parasitemia was measured at day 12 and 14 post-infection, *p=0.0182, Mann-Whitney test, (Data are expressed as means ± standard deviations). **(C, D)** After Dm28c *T. cruzi* infection of C57BL/6 mice treated with PBS or double 5FU TSf-ISPA mice, parasitemia was measured at day 12 and 14 post-infection (data are expressed as means ± standard deviations) (n = 5 per group).

To analyze whether the vaccine formulation has protective capacity against additional DTUs, i.p. infection with Dm28c (DTU I) was performed. This strain does not commonly cause mortality during acute infection and generates lower parasitemia levels. After the immunization protocol, both strains BALB/c and C57BL/6 mice were i.p. infected with 20000 cultured trypomastigotes. As expected, there was no difference in the percentage of survival, given that control and infected BALB/c and C57BL/6 mice survived 100% after infection. However, results obtained showed that parasitemia levels were significantly reduced in vaccinated BALB/c mice at day 14 post-infection (**Figure 13B**), as compared to infected PBS-treated mice, and showed a similar trend at day 12 post-infection in BALB/c mice, and at day 12 and 14 post-infection in vaccinated and control C57BL/6 mice (**Figures 13A, C, D**).

**Figure 14:**
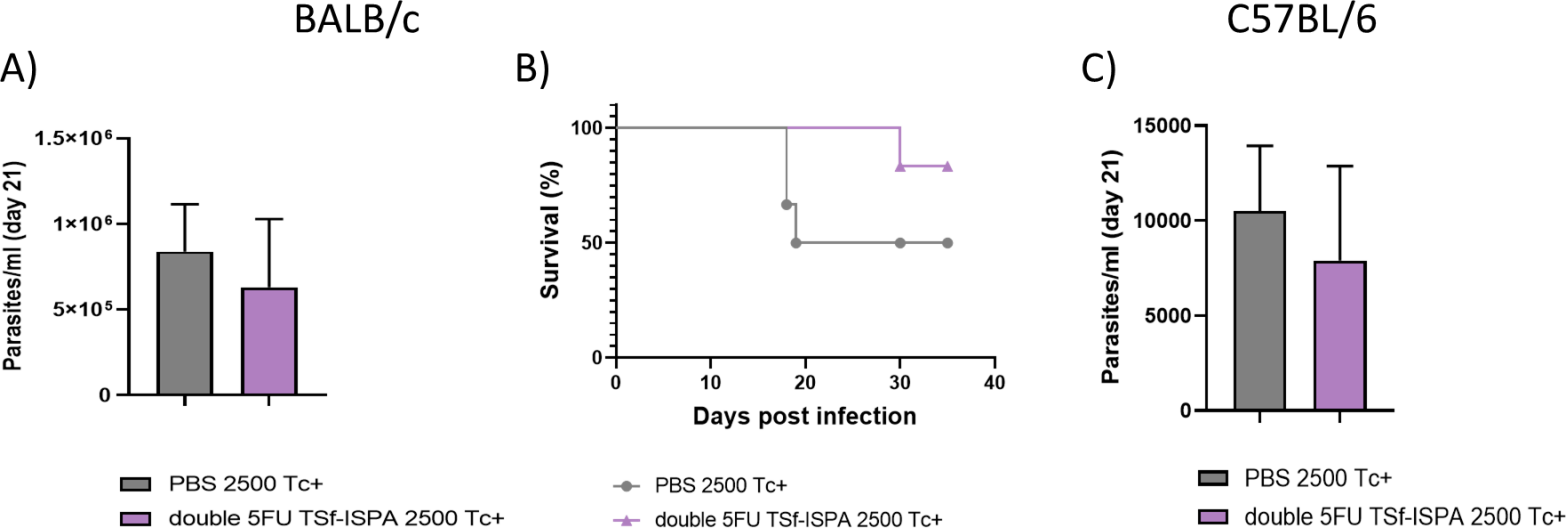
Parasitemia and survival of BALB/c and C57BL/6 mice treated with double 5FU TSf-ISPA and intradermically infected with Tulahuen *T. cruzi* parasites. Mice were treated with PBS or double 5FU TSf-ISPA mice and were intradermically infected with 2500 trypomastigotes Tulahuen fifteen days after the last immunization dose. **(A)** Parasitemia of PBS and double 5FU TSf-ISPA immunized BALB/c mice at day 21 post-infection (data are expressed as means ± standard deviations). **(B)** Survival is shown until day 35 post-infection of BALB/c mice. **(C)** Parasitemia of PBS and double 5FU TSf-ISPA immunized C57BL/6 mice at day 21 post-infection (data are expressed as means ± standard deviations) (n=5 per group).

Then, to analyze a model of infection resembling a natural route of *T. cruzi* transmission, i.d. infection was accomplished in BALB/c and C57BL/6 mice with the Tulahuen strain, which generates a more evident effect. After i.d. infection with 2500 trypomastigotes, double 5FU TSf-ISPA vaccinated BALB/c mice showed lower parasitemia levels at day 21 post-infection (**Figure 14A**) and a clear increase in survival (**Figure 14B**) compared to PBS-treated and infected mice.

In addition, a clear trend suggesting protection was also observed in C57BL/6 mice at day 21 post-infection of double 5FU TSf-ISPA compared to PBS-control mice (**Figure 14C**). Thus, taken together, these results provide strong evidence supporting that the double 5FU TSf-ISPA vaccine can confer protection in several different preclinical models, including different genetic backgrounds of the host, different DTUs of parasites, and different doses and routes of *T. cruzi* infection.

## Discussion

Many successful pathogens have evolved complex strategies to cope with the host immune system. Consequently, the development of vaccines may require adapting and advancing the strategies employed to control such types of microorganisms. This approach would benefit from comprehensive consideration of the whole immune system, encompassing not only the usual priming of effector immune responses, but also targeting the regulatory arm to level-up vaccine development.

Regarding *T. cruzi*, a successful vaccine is expected to meet at least some requirements, including production of neutralizing and trypanolytic antibodies, generation of memory cells targeting infected cells, elicitation of a response capable to interfere with the well-known manipulation of the host immune system, and generation of cross-protective capacity against parasites from different DTUs. Since the TS enzyme from *T. cruzi* is a promising antigen which potential capacity to fulfill all these requirements (12–14, 17, 18), a TS-based antigen, together with an adjuvant (ISPA) able to elicit a cellular immune response (28) were selected as the basis of our formulation, from the initial stages of vaccine development (26).

The TSf-ISPA vaccine candidate showed a significant protective capacity in previous studies (25, 26). In addition, since marked alterations of MDSCs were registered during both the immunization and the infection phases, the aim of this work was focused into rationally control MDSC involvement during TSf-ISPA immunization, to further potentiate a vaccine protocol with protective capacity in multiple mouse models.

Results obtained herein showed that Gr-1+ cells that increase after TSf-ISPA immunization have suppressive capacity, as measured using standard *in vitro* assays. This result adds evidence and aligns with recent data supporting that the increase of MDSCs during immunization is not an unexpected result or an exception, but instead it is a common mechanism associated to many protocols that differ in immunogens, adjuvants, and immunization routes (27, 51–54).

In this case, purified Gr-1+ splenocytes from TSf-ISPA vaccinated mice showed suppressive capacity over BMDCs and CD4+ lymphocytes. Although the analysis of suppressive function over lymphocytes has been commonly used to demonstrate the involvement of MDSCs, results described herein provide evidence that measuring suppressive capacity over DCs may also be very useful, particularly given the significant difference observed regarding the inhibition of DC maturation markers in the presence of MDSCs. Moreover, a recent report has also described that MDSCs´ suppression may even be strong enough to kill DCs, as reported in a model of immunization with heat-killed *M. tuberculosis* (54).

Despite several studies have reported that immunization may cause MDSCs increases, to our knowledge, a deep analysis of the kinetics of the cells has not been previously addressed. For this purpose, the alterations of MDSCs were analyzed during a two-week follow-up period after vaccination with TSf-ISPA.

This analysis was useful to establish the temporal expansion of MDSCs, which increased very early at day 2 post-imm. with TSf-ISPA, then remained increased at day 7, and finally contracted by day 15 post-imm. Interestingly, MDSC depletion with 5FU the day before each dose of the vaccine was successful to deplete the early increase of MDSCs, but this depletion was transient and failed to maintain lower levels of those cells after immunization. During 5FU TSf-ISPA vaccination, MDSCs also increased by day 7 post-imm., likely as a rebound effect (55), and then slightly contracted by day 15 post-imm. Even if the early depletion of MDSCs correlated with better protection, as we have previously reported (25), the fact that MDSCs still increased by day 7 post-imm. opened the possibility to further optimize the vaccine protocol adding a second dose of 5FU at day 8 post-imm. to target the MDSCs that augmented by day 7 post-imm. This new optimized protocol was successful to early deplete MDSCs at day 2, and to completely deplete MDSCs by day 15 post-imm. These findings on the impact of 5FU on MDSC kinetics may prove valuable for rational vaccine design in general, but especially when considering vaccines known to increase MDSCs in various immunization settings, where they can limit vaccine efficacy (27).

To experimentally prove whether the depletion of MDSCs with 2 doses of 5FU was able to improve TSf-ISPA protective capacity, infection assays were conducted. Since 5FU TSf-ISPA vaccinated mice already showed a 100% survival against i.p. infection with a lethal dose of 1500 Tulahuen parasites (25), in this study, very high lethal doses of 2000 and 3000 parasites were used to cause some grade of mortality in vaccinated mice. As expected, 100% of PBS-treated control mice succumbed very early after the challenge and with high parasitemia levels. In addition, mice that received two doses of 5FU showed the best performance regarding parasitemia and survival against both challenges of parasites, strongly supporting that 2 dose of 5FU further improves the vaccine protocol based on the TSf-ISPA formulation.

These results are in line with several studies reporting that MDSCs increases during the immunization phase may limit the vaccine efficacy against several pathogens (27, 51–53). On the other hand, it has been reported that MDSC increases during immunization with *Candida* species may have a protective role during infection (56). The fact that double 5FU TSf-ISPA completely decreased MDSCs before infection suggests that, at least in our model, MDSCs were not playing a protective role.

Moreover, this observation also aligns with the fact that, during infection, lower levels of MDSCs correlated with better protection after infection of TSf-ISPA immunized mice as compared to PBS-treated mice (26). Further supporting all this data, results reported herein showed that double 5FU TSf-ISPA vaccinated mice have lower absolute number of MDSCs than 5FU TSf-ISPA mice, correlating with better outcome and thus providing additional evidence that lower levels of MDSCs after infection favors a stronger immune effector response in vaccinated mice.

To analyze whether in fact the second dose of 5FU allowed the elicitation of a higher immune effector response, several immune populations were studied at day 7 post-imm. As showed in this study, after 3 dose of immunizations, double 5FU TSf-ISPA immunized mice showed higher absolute numbers of CD4+, CD8+, CD4+ CD44+ CD8+ CD44+, CD11c+ and CD11c CD8α+ cells in both spleen and lymph nodes as compared with the animals that received 5FU TSf-ISPA. Moreover, cultured splenocytes from double 5FU TSf-ISPA mice showed a higher percentage of IFN-γ+ lymphocytes and greater production of IFN-γ in the supernatants compared to 5FU TSf-ISPA mice. Thus, the protocol based on 2 doses of 5FU showed complete depletion of MDSCs at day 15 post-imm., caused higher increases of several immune populations during vaccination, and then correlated with lower levels of MDSCs and better outcome of the infection. Taken together, our previous reports and the data presented herein support that MDSCs are markedly involved both during immunization against *T. cruzi* and after infection. Depleting MDSCs during immunization may allow the elicitation of a stronger effector immune response against the parasite, in a way that enables better control post-infection, preventing the excessive increase of MDSCs that suppress the immune response and benefit the parasite.

It is to note that additional protocols were assayed during the optimization of this vaccine protocol. For instance, the administration of 5FU the same day of the TSf-ISPA vaccine generated a slightly lower response than the protocol administrating the 5FU the day before TSf-ISPA immunization (data not shown). Considering the protocol with the highest efficacy, the administration of 5FU before TSf-ISPA vaccine was selected for this work. However, the possibility exists to also continue improving the protocol based on the administration of 5FU and TSf-ISPA simultaneously, a fact that would favor practical implementation. In addition, the second dose of 5FU was also assayed at day 4 post-imm., but this approach did not improve the protective capacity (data not shown), suggesting that 2 doses separated by 7 days is preferable, and that protocol was selected as the more effective.

As previously mentioned, the genetic diversity of the parasite may also pose another potential obstacle to advancing toward a successful vaccine against *T. cruzi*. Since it has been described that there is a high identity between TSs from different DTU, TS-based immunogens have been suggested as promising antigens to generate protection against different DTUs (12). Despite this *in silico* prediction, experimental assessment of the cross-protective capacity of TSs in different preclinical mouse models has scarcely been performed (3, 11).

Thus, after selecting an optimized vaccine protocol, to further advance in the preclinical assessment of our vaccine candidate, the protective capacity of double 5FU TSf-ISPA vaccine was assessed in several mouse models, involving different DTUs, host strains, parasites doses, and routes of infection.

First, a general analysis was accomplished in C57BL/6 mice, that had not been used previously in the assessment of the TSf-ISPA vaccine candidate. Despite the differences in the genetic background, results described herein showed that the double 5FU TSf-ISPA vaccine protocol generated a marked effector immune response in C57BL/6 mice, as measured by antibodies levels and DTH response. A standard dose of 1000 Tulahuen *T. cruzi* administered i.p. did not cause differences in survival, but showed a trend of better protection regarding parasitemia in vaccinated mice as compared to PBS-treated control mice. Moreover, vaccinated mice also showed lower levels of MDSCs than control mice, an event that correlates with better protection in previous studies (25, 26) and with other experiments described herein.

Dm28c is a *T. cruzi* strain belonging to DTU I. It has been described that this strain does not generally cause high mortality during acute infection of mice (57). Despite a difference in the protective capacity could be difficult to be observed according to the characteristics of the Dm28c strain, the experiments performed showed a clear trend suggesting that both BALB/c and C57BL/6 vaccinated mice were able to better control parasitemia after i.p. *T. cruzi* infection, as compared to PBS-treated control mice. Also supporting the results presented herein, **Supplementary Figure 7** shows that male BALB/c mice vaccinated with 5FU TSf-ISPA had better protective capacity against the high virulent RA strain (DTU VI). After i.p. infection, parasitemia was lower and survival was higher in vaccinated mice compared to control mice, even without using the double protocol based on two dose of 5FU, adding evidence that TSf-ISPA vaccine, potentiated by the 5FU treatment confer protection in multiple mouse models of *T. cruzi* infection.

Finally, since i.d. inoculation resembles one of the most important routes of transmission, this via was used to accumulate more evidence of the protective capacity of the optimized vaccine protocol. Tulahuen parasites were used, considering that this strain causes more evident effects. Once again, double 5FU TSf-ISPA vaccinated mice showed significant lower parasitemia than PBS-treatment after infection of BALB/c and C57BL/6 mice and a clear trend supporting that survival is also increased in vaccinated BALB/c mice after i.d. infection with 2500 parasites.

Thus, taking together, results reported herein supports that the vaccine protocol based on double 5FU TSf-ISPA immunization effectively generates cross-protection in several mouse models of *T. cruzi* infection.

It has been described that TSs can elicit CD8 immunodominant responses targeting particular epitopes encoded by the TS genes (58). It remains to be solved whether this characteristic is beneficial or detrimental for the parasite and for vaccine development (58–60). In this sense, some reports have described that a vaccine based only in one immunodominant epitope is able to generate protection against *T. cruzi*, whereas the addition of subdominant epitopes decreased the effectivity of the vaccine (61). On the other hand, it has also been suggested that the immunodominant epitopes of TSs are not critical for parasite infectivity nor for vaccine development (58), and there is evidence showing that subdominant epitopes are also important for resistance (60). Since TSf immunogen used in this study lacks the sequence that would acts as the most immunodominant epitope (IYNVGQVSI) in BALB/c mice and does not have an immunodominant epitope in C57BL/6 mice, our results support that immunodominant epitopes are not indispensable to develop a vaccine with cross-protective capacity in multiple mouse models of *T. cruzi* infection.

Vaccines are one of the most successful advances in public health. Nonetheless, there are many diseases caused by complex pathogens that are still elusive to prophylactic prevention or therapeutic treatment with licensed vaccines. Results described herein provide evidence that rationally controlling the involvement of cells from the regulatory arm of the immune system potentiates vaccine efficacy against *T. cruzi*. Further studies are warranted to continue maximizing vaccine protection. The generation of complementary strategies to level up traditional approaches may favor the development of the vaccines that are still lacking.

## Data Availability

The raw data supporting the conclusions of this article will be made available by the authors, without undue reservation.
