# Supplementary material for: Control of myeloid-derived suppressor cell dynamics potentiates vaccine protection in multiple mouse models of Trypanosoma cruzi infection
Source: Front Immunol. 2024 Nov 1;15:1484290. doi: 10.3389/fimmu.2024.1484290 (PMC11568482; doi:10.3389/fimmu.2024.1484290)
Supplement: Supplementary file 1 [file DataSheet1.pdf]

Supplementary Table I

Group	Conserved motifs	Functions	Enzymatic activity	Antigenicity
Group I	VTVxNVxLYNR, FRIP, Asp-box.	Active trans-sialidases (sialic acid transfer).	Yes	Yes
Group II	VTVxNVxLYNR, Asp-box, without FRIP.	Cell adhesion and invasion.	No	Yes
Group III	VTVxNVxLYNR, FRIP, without Asp-box).	Complement regulation.	No	Yes
Group IV	VTVxNVxLYNR FRIP, Asp-box	Unknown function.	No	Yes
Group V	VTVxNVxLYNR, Asp-box, without FRIP.	Unknown function.	No	Yes
Group VI	VTVxNVxLYNR Asp-box, without FRIP.	Unknown function.	No	Yes
Group VII	VTVxNVxLYNR, FRIP, without Asp-box.	Potential regulatory function of complement.	No	Yes
Group VIII	VTVxNVxLYNR, FRIP, without Asp-box.	Unknown function.	No	Yes
