# Supplementary material for: Control of myeloid-derived suppressor cell dynamics potentiates vaccine protection in multiple mouse models of Trypanosoma cruzi infection
Source: Front Immunol. 2024 Nov 1;15:1484290. doi: 10.3389/fimmu.2024.1484290 (PMC11568482; doi:10.3389/fimmu.2024.1484290)
Supplement: Supplementary file 2 [file DataSheet2.pdf]

Figure			Mean	SD	test	p-value	More than two groups			
							Difference intercolumn	Shapiro-Wilk normality test	Levene Test of Variances	Observation
1	% MHCII high cells within CD11chigh+ cells	CDs<br>CDs+MDSCs	64.5<br>48.15	7.3<br>9.5	t- test one tail	0.0288		Normality cannot be discarded	Homoscedasticity cannot be discarded	A one-tailed test was used because it has been reported previosuly that MDSCs can decrease DC activation markers.
	MFI MHCII cells within CD11chigh+ cells	CDs<br>CDs+MDSCs	181115<br>102897	34873<br>26137	Mann-Whitney one tail	0.0288		Normality discarded in one group		
	% CD80high cells within CD11c high+ cells	CDs<br>CDs+MDSCs	78.27<br>56.5	5.054<br>7.248	t- test one tail	0.0288		Normality cannot be discarded	Homoscedasticity cannot be discarded	
	MFI CD80 cells within CD11chigh+ cells	CDs<br>CDs+MDSCs	230654<br>129049	28833<br>22873	t- test one tail	0.0288		Normality cannot be discarded	Homoscedasticity cannot be discarded	
2	Control<br>Control + MDSCs		47.2<br>14.13	3.341<br>1.401	t- test	< 0.001		Normality cannot be discarded	Homoscedasticity cannot be discarded	A one-tailed test was used because it has been reported previosuly MDSCs can decrease DC activation markers.
3	Day 2<br>N abs CD11b+ Gr-1+ cells	PBS<br>TSf-ISPA	3.4<br>6.7	1.058<br>1.145	t- test	0.0015		Normality cannot be discarded	Homoscedasticity cannot be discarded	A one-tailed test was used because previous data indicated an increase in MDSCs during immunization
	Day 7<br>N abs CD11b+ Gr-1+ cells	PBS<br>TSf-ISPA	2.85<br>6.667	0.2121<br>1.79	Mann-Whitney one tail	0.1		Normality cannot be discarded	Homoscedasticity discarded	
	Day 15<br>N abs CD11b+ Gr-1+ cells	PBS<br>TSf-ISPA	2.65<br>4.633	0.2121<br>2.312	Mann-Whitney one tail	0.1		Normality cannot be discarded	Homoscedasticity discarded	
4	Day 2<br>N abs CD11b+ Gr-1+ cells	PBS<br>5FU TSf-ISPA	31.525<br>3.092	0.71<br>1.47	t- test	ns		Normality cannot be discarded	Homoscedasticity cannot be discarded	A two-tailed test was used because it was not possible to infer the direction of the change
	Day 7<br>N abs CD11b+ Gr-1+ cells	PBS<br>5FU TSf-ISPA	4.08<br>15.66	1.03<br>8.3	t- test	0.0149		Normality cannot be discarded	Homoscedasticity cannot be discarded	
	Day 15<br>N abs CD11b+ Gr-1+ cells	PBS<br>5FU TSf-ISPA	2.87<br>14.3	0.321<br>1.235	t- test	<0.001		Normality cannot be discarded	Homoscedasticity cannot be discarded	

5 A	Day 2<br>N abs CD11b+ Gr-1+ cells	PBS<br>double 5FU TSf-ISPA	2.808<br>3.682	0.990<br>1.69	t- test	ns		Normality cannot be discarded	Homoscedasticity cannot be discarded	A two-tailed test was used because it was not possible to infer the direction of the change
	Day 7<br>N abs CD11b+ Gr-1+ cells	PBS<br>double 5FU TSf-ISPA	3.98<br>16.66	0.9558<br>5.27	t- test	0.0008		Normality cannot be discarded	Homoscedasticity cannot be discarded	
	Day 15<br>N abs CD11b+ Gr-1+ cells	PBS<br>double 5FU TSf-ISPA	3.138<br>3.36	1.22<br>1.79	t- test	ns		Normality cannot be discarded	Homoscedasticity cannot be discarded	
5 B	Day 2<br>Ratio n abs CD11b+ Gr-1+	A TSf-ISPA<br>B 5FU TSf-ISPA<br>C double 5FU TSf-ISPA	1.98<br>0.986<br>1.164	0.35<br>0.45<br>0.53	ANOVA	0.0081	A vs B p-value 0.0032<br>A vs C p-value 0.0139<br>B vs C ns	Normality cannot be discarded	Homoscedasticity cannot be discarded	
	Day 7<br>Ratio n abs CD11b+ Gr-1+	A TSf-ISPA<br>B 5FU TSf-ISPA<br>C double 5FU TSf-ISPA	2.333<br>4.25<br>4.04	0.56<br>1.55<br>1.3	ANOVA	ns	A vs B ns<br>A vs C ns<br>B vs C ns	Normality cannot be discarded	Homoscedasticity cannot be discarded	
	Day 15<br>Ratio n abs CD11b+ Gr-1+	A TSf-ISPA<br>B 5FU TSf-ISPA<br>C double 5FU TSf-ISPA	1.76<br>3.44<br>0.97	0.89<br>1.14<br>0.5	ANOVA	0.0043	A vs B p-value 0.0277<br>A vs C ns<br>B vs C p-value 0.0014	Normality cannot be discarded	Homoscedasticity cannot be discarded	
6	Parasites/ml	5FU TSf-ISPA<br>double 5FU TSf-ISPA	22195<br>12305	13260<br>8403	Mann-Whitney	ns		Normality discarded in one group		
	Parasites/ml	5FU TSf-ISPA<br>double 5FU TSf-ISPA	42933<br>4842	9662<br>2603	Mann-Whitney	0.0357		Normality discarded in one group		

7	Antibodies	A	PBS	0.07667	0.0012	ANOVA	<0.0001	A vs B p-value <0.0001	Normality cannot be discarded	Homoscedasticity cannot be discarded	
		B	TSf-ISPA	0.6862	0.1022			A vs C p-value<0.0001			
		C	5FU TSf-ISPA	0.7829	0.1409			A vs D P-value <0.0001			
		D	double 5FU TSf-ISPA	0.8367	0.1624						
	DTH	A	PBS	0.04	0.4648	Kruskal-Wallis	0.0001	A vs C p-value 0.0071	Normality discarded		
		B	TSf-ISPA	0.475	0.09574			A vs D p-valu <0.0001			
		C	5FU TSf-ISPA	0.5875	0.1126			B vs D p-value 0.018			
		D	double 5FU TSf-ISPA	0.75	0.1354						
8	N abs CD4+ cells	A	PBS	9.66	2.22	ANOVA	<0.0001	A vs B p-value 0.0091	Normality cannot be discarded	Homoscedasticity cannot be discarded	
		B	5FU TSf-ISPA	17.5	2.94			A vs C p-valu <0.0001			
		C	double 5FU TSf-ISPA	28.4	5.849			B vs C p-value 0.001			
	N abs CD8+ cells	A	PBS	2.78	0.7014	ANOVA	<0.0001	A vs B p-value 0.0126	Normality cannot be discarded	Homoscedasticity cannot be discarded	
		B	5FU TSf-ISPA	5.184	1.276			A vs C p-valu <0.0001			
		C	double 5FU TSf-ISPA	8.62	1.712			B vs C p-value 0.0013			
	N Abs CD4+ CD44+ cells	A	PBS	4.09	0.9463	ANOVA	0.0002	A vs B p-value 0.0175	Normality cannot be discarded	Homoscedasticity cannot be discarded	
		B	5FU TSf-ISPA	6.18	0.5541			A vs C p-valu <0.0001			
		C	double 5FU TSf-ISPA	8.74	1.766			B vs C p-value 0.0055			
	N Abs CD8+ CD44+ cells	A	PBS	0.776	0.1806	ANOVA	0.0006	A vs B p-value 0.213	Normality cannot be discarded	Homoscedasticity cannot be discarded	
		B	5FU TSf-ISPA	1.288	0.3355			A vs C p-valu 0.0002			
		C	double 5FU TSf-ISPA	1.814	0.3677			B vs C p-value 0.0186			

8	N abs CD11c+ cells	A	PBS	0.778	0.2725	ANOVA	ns		Normality cannot be discarded	Homoscedasticity cannot be discarded
		B	5FU TSf-ISPA	1.243	0.5731					
		C	double 5FU TSf-ISPA	1.314	0.4571					
	N abs CD8+ CD11c+ cells	A	PBS	0.1012	0.02175	ANOVA	0.0245	A vs C<br>p-valu 0.0082	Normality cannot be discarded	Homoscedasticity cannot be discarded
		B	5FU TSf-ISPA	0.1428	0.03419					
		C	double 5FU TSf-ISPA	0.216	0.09072					
	% CD4+ IFN+ cells	A	PBS	0.2725	0.0562	ANOVA	<0.0001	A vs C<br>p-valu <0.0001<br>B vs C<br>p-value <0.0001	Normality cannot be discarded	Homoscedasticity cannot be discarded
		B	5FU TSf-ISPA	0.29	0.06782					
		C	double 5FU TSf-ISPA	0.948	0.2395					
	% CD8+ IFN+ cells	A	PBS	0.023	0.01249	ANOVA	0.0829	A vs C<br>p-valu 0.0392	Normality cannot be discarded	Homoscedasticity cannot be discarded
		B	5FU TSf-ISPA	0.032	0.01503					
		C	double 5FU TSf-ISPA	0.0656	0.03881					
	IFN pg/ml	A	PBS	n.d	n.d	ANOVA	0.0003	A vs C<br>p-valu 0.0002<br>B vs C<br>p-value 0.0003	Normality cannot be discarded	Homoscedasticity cannot be discarded
		B	5FU TSf-ISPA	303.3	122.4					
		C	double 5FU TSf-ISPA	2708	684.6					
9	N abs CD4+ cells	A	PBS	1.377	0.1762	ANOVA	<0.0001	A vs B<br>p-value 0.0016<br>A vs C<br>p-valu <0.0001<br>B vs C<br>p-value 0.009	Normality cannot be discarded	Homoscedasticity cannot be discarded
		B	5FU TSf-ISPA	2.747	0.1361					
		C	double 5FU TSf-ISPA	4.1	0.4848					
	N abs CD8+ cells	A	PBS	0.4067	0.1041	ANOVA	<0.0001	A vs B<br>ns<br>A vs C<br>p-valu <0.0001<br>B vs C<br>p-value <0.0001	Normality cannot be discarded	Homoscedasticity cannot be discarded
		B	5FU TSf-ISPA	0.6	0.1					
		C	double 5FU TSf-ISPA	1.28	0.1304					

9	N Abs CD4+ CD44+ cells	A<br>B<br>C	PBS<br>5FU TSf-ISPA<br>double 5FU TSf-ISPA	0.16<br>0.4767<br>0.576	0.03606<br>0.1012<br>0.08961	ANOVA	0.0004	A vs B p-value 0.0016<br>A vs C p-value 0.0001<br>B vs C ns	Normality cannot be discarded	Homoscedasticity cannot be discarded	
	N Abs CD8+ CD44+ cells	A<br>B<br>C	PBS<br>5FU TSf-ISPA<br>double 5FU TSf-ISPA	0.08167<br>0.1533<br>0.35	0.02566<br>0.02309<br>0.09487	ANOVA	0.0015	A vs C p-valu 0.0007<br>B vs C p-value 0.0046	Normality cannot be discarded	Homoscedasticity cannot be discarded	
	N abs CD11c+ cells	A<br>B<br>C	PBS<br>5FU TSf-ISPA<br>double 5FU TSf-ISPA	0,046<br>0.112<br>0.212	0,005292<br>0.01058<br>0.03633	ANOVA	<0.0001	A vs B p-value 0.0154<br>A vs C p-valu <0.0001<br>B vs C p-value 0.0008	Normality cannot be discarded	Homoscedasticity cannot be discarded	
	N abs CD8+ CD11c+ cells	A<br>B<br>C	PBS<br>5FU TSf-ISPA<br>double 5FU TSf-ISPA	0.014<br>0.03167<br>0.0662	0.004583<br>0.002309<br>0.009935	ANOVA	<0.0001	A vs B p-value 0.0201<br>A vs C p-valu <0.0001<br>B vs C p-value 0.0002	Normality cannot be discarded	Homoscedasticity cannot be discarded	
10	Abs n CD11b+ Ly6G+ Ly6C+/low cells (x106)		5FU TSf-ISPA<br>double 5FU TSf-ISPA	64.39<br>38.73	9.785<br>3.601	t-test one tail	0.0163		Normality cannot be discarded	Homoscedasticity discarded	A one-tailed test was used because better protection has been shown to correlate with lower MDSC levels
	Abs n CD11b+ Ly6C+ Ly6G- cells (x106)		5FU TSf-ISPA<br>double 5FU TSf-ISPA	10.56<br>4.025	5.632<br>1.477	Mann-Whitney one tail	0.0286		Normality cannot be discarded	Homoscedasticity discarded	A one-tailed test was used because better protection has been shown to correlate with lower MDSC levels

11	Antibodies	PBS<br>double 5FU TSf-ISPA	0.1102<br>0.8103	0.04933<br>0.6041	Mann-Whitney one tail	0.0022		Normality cannot be discarded	Homoscedasticity discarded	
	DTH	PBS<br>double 5FU TSf-ISPA	0.0125<br>0.04167	0.004183<br>0.007528	Mann-Whitney one tail	0.0011		Normality discarded		
12	Abs n CD11b+ Ly6G+ Ly6C+/low	A NI<br>B double 5FU TSf-ISPA<br>C PBS	1.15<br>21.52<br>55.6	0.05<br>3.343<br>28.98	Anova	<0.0001	A vs C p-valu 0.0009<br>B vs C p-value 0.0201	Normality cannot be discarded	Homoscedasticity cannot be discarded	
	Abs n CD11b+ Ly6G- Ly6C+	A NI<br>B double 5FU TSf-ISPA<br>C PBS	1.753<br>9.042<br>22.52	0.255<br>1.684<br>9.564	Anova	0.001	A vs C p-valu 0.0005<br>B vs C p-value 0.0029	Normality cannot be discarded	Homoscedasticity cannot be discarded	
13	Parasites/ml Day 12 BALB/c	PBS<br>double 5FU TSf-ISPA	647<br>287.3	491.1<br>266.3	Mann-Whitney	0.1		Normality discarded		
	Parasites/ml Day 14 BALB/c	PBS<br>double 5FU TSf-ISPA	934.3<br>179.5	756<br>213.8	Mann-Whitney	0.0182		Normality discarded		
	Parasites/ml Day 12 C57BL/6	PBS<br>double 5FU TSf-ISPA	153,3<br>76.67	268.9<br>118.8	Mann-Whitney	ns		Normality discarded		
	Parasites/ml Day 14 C57BL/6	PBS<br>double 5FU TSf-ISPA	191.7<br>76.67	168.9<br>118.8	Mann-Whitney	ns		Normality discarded		
14	Parasites/ml Day 21 BALB/c	PBS<br>double 5FU TSf-ISPA	10494<br>7891	3456<br>4983	Mann-Whitney	ns		Normality discarded		
	Parasites/ml Day 21 C57BL/6	PBS<br>double 5FU TSf-ISPA	144<br>72.5	128.4<br>79.42	Mann-Whitney	ns		Normality discarded		

Supplementary figures			Mean	SD	test	p-value	More than two groups			
							Difference intercolumn	Shapiro-Wilk normality test	Levene Test of Variances	Observation
2 A	Percentage CD11b+ Gr-1+ cells Day 2	PBS<br>TSf-ISPA	3.79<br>6.68	1.15<br>0.65	t- test one tail	0.001		Normality cannot be discarded	Homoscedasticity cannot be discarded	A one-tailed test was used because previous data indicated an increase in MDSCs during immunization
	Percentage CD11b+ Gr-1+ cells Day 7	PBS<br>TSf-ISPA	3.5<br>6.53	0.49<br>0.65	t- test one tail	0.0059		Normality cannot be discarded	Homoscedasticity cannot be discarded	
	Percentage CD11b+ Gr-1+ cells Day 15	PBS<br>TSf-ISPA	3.7<br>4.87	0.56<br>0.58	t- test	ns		Normality cannot be discarded	Homoscedasticity cannot be discarded	
2 B	Percentage CD11b+ Gr-1+ cells Day 2	PBS<br>5FU TSf-ISPA	4.925<br>6.05	2.11<br>2.70	t- test one tail	ns		Normality cannot be discarded	Homoscedasticity cannot be discarded	
	Percentage CD11b+ Gr-1+ cells Day 7	PBS<br>5FU TSf-ISPA	4.704<br>13.35	0.55<br>2.12	t- test two tail	0.0002		Normality cannot be discarded	Homoscedasticity cannot be discarded	
	Percentage CD11b+ Gr-1+ cells Day 15	PBS<br>5FU TSf-ISPA	5.12<br>11.625	0.82<br>0.97	t- test two tail	0.0013		Normality cannot be discarded	Homoscedasticity cannot be discarded	
2 C	Percentage CD11b+ Gr-1+ cells Day 2	PBS<br>double 5FU TSf-ISPA	3.5<br>4.76	0.8<br>0.7	t- test two tail	ns		Normality cannot be discarded	Homoscedasticity cannot be discarded	
	Percentage CD11b+ Gr-1+ cells Day 7	PBS<br>double 5FU TSf-ISPA	4.375<br>14.03	0.39<br>4.91	t- test two tail	0.0023		Normality cannot be discarded	Homoscedasticity cannot be discarded	
	Percentage CD11b+ Gr-1+ cells Day 15	PBS<br>double 5FU TSf-ISPA	3.905<br>4.12	1.37<br>2.1	t- test two tail	ns		Normality cannot be discarded	Homoscedasticity cannot be discarded	

2 D	Ratio percentage CD11b+ Gr-1+ cells Day 2	A<br>B<br>C	TSf-ISPA<br>5FU TSf-ISPA<br>double 5FU TSf-ISPA	1.748<br>0.958<br>13.825	0.18<br>0.39<br>017	ANOVA	0.011	A vs B p-value 0.0038<br>A vs C p-value 0.0301<br>B vs C ns	Normality cannot be discarded	Homoscedasticity cannot be discarded	
	Ratio percentage CD11b+ Gr-1+ cells Day 7	A<br>B<br>C	TSf-ISPA<br>5FU TSf-ISPA<br>double 5FU TSf-ISPA	1.9<br>2,68<br>3.162	0.2<br>1.09<br>1.09	ANOVA	ns		Normality cannot be discarded	Homoscedasticity cannot be discarded	
	Ratio percentage CD11b+ Gr-1+ cells Day 15	A<br>B<br>C	TSf-ISPA<br>5FU TSf-ISPA<br>double 5FU TSf-ISPA	1.3<br>2.42<br>0.856	0.17<br>0.37<br>0.53	ANOVA	0.0007	A vs B p-value 0.0061<br>A vs C ns<br>B vs C p-value 0.0002	Normality cannot be discarded	Homoscedasticity cannot be discarded	
3	% CD11b+ Ly6G+ Ly6C+/low cells	A<br>B<br>C<br>D	PBS<br>TSf-ISPA<br>5FU TSf-ISPA<br>double 5FU TSf-ISPA	1.2<br>3.1<br>6.25<br>11.1	0.11<br>1.8<br>1.94<br>3.0	ANOVA	0.0009	A vs B 0.093<br>A vs C 0.0002<br>B vs C 0.008<br>B vs D 0.01	Normality cannot be discarded	Homoscedasticity cannot be discarded	
	% CD11b+ Ly6C+ Ly6G- cells	A<br>B<br>C<br>D	PBS<br>TSf-ISPA<br>5FU TSf-ISPA<br>double 5FU TSf-ISPA	0.3<br>0.61<br>0.84<br>1.52	0.025<br>0.18<br>0.059<br>0.33	ANOVA	<0.0001	A vs C 0.0038<br>A vs D 0<0.001<br>B vs C 0.0002<br>B vs D 0.0008	Normality cannot be discarded	Homoscedasticity cannot be discarded	
	Abs n CD11b+ Ly6G+ Ly6C+/low cells	A<br>B<br>C<br>D	PBS<br>TSf-ISPA<br>5FU TSf-ISPA<br>double 5FU TSf-ISPA	0.95<br>3.31<br>13.05<br>18.1	0.05<br>1.9<br>4.7<br>4.9	ANOVA	0.0002	A vs C 0.0019<br>A vs D <0.0001<br>B vs C 0.0007<br>B vs D 0.0008	Normality cannot be discarded	Homoscedasticity cannot be discarded	
	Abs n CD11b+ Ly6C+ Ly6G- cells	A<br>B<br>C<br>D	PBS<br>TSf-ISPA<br>5FU TSf-ISPA<br>double 5FU TSf-ISPA	0.24<br>0.62<br>1.83<br>2.4	0.02<br>0.87<br>0.16<br>0.53	ANOVA	0.0101	A vs C <0.0001<br>A vs D <0.0001<br>B vs C 0.0002<br>B vs D <0.0001	Normality cannot be discarded	Homoscedasticity cannot be discarded	

4	% CD4+ cells	A<br>B<br>C	PBS<br>5FU TSf-ISPA<br>double 5FU TSf-ISPA	11.98<br>13.02<br>24.34	2.339<br>2.804<br>2.002	ANOVA	< 0.0001	A vs C p-value <0.0001<br>B vs C p-value <0.0001	Normality cannot be discarded	Homoscedasticity cannot be discarded	
	% CD8+ cells	A<br>B<br>C	PBS<br>5FU TSf-ISPA<br>double 5FU TSf-ISPA	3.46<br>3.892<br>7.376	0.7829<br>1.2<br>0.4986	ANOVA	< 0.0001	A vs C p-value <0.0001<br>B vs C p-value <0.0001	Normality cannot be discarded	Homoscedasticity cannot be discarded	
	% CD4+ CD44+ cells	A<br>B<br>C	PBS<br>5FU TSf-ISPA<br>double 5FU TSf-ISPA	5.098<br>4.564<br>7.502	1.117<br>0.5736<br>0.658	ANOVA	0.0002	A vs C p-value 0.0006<br>B vs C p-value 0.0001	Normality cannot be discarded	Homoscedasticity cannot be discarded	
	%CD8+ CD44+ cells	A<br>B<br>C	PBS<br>5FU TSf-ISPA<br>double 5FU TSf-ISPA	0.972<br>0.956<br>1.548	0.221<br>0.273<br>0.1984	ANOVA	0.0023	A vs C p-value 0.0021<br>B vs C p-value 0.0017	Normality cannot be discarded	Homoscedasticity cannot be discarded	
	% CD11c+ cells	A<br>B<br>C	PBS<br>5FU TSf-ISPA<br>double 5FU TSf-ISPA	1.016<br>0.806<br>0.922	0.04879<br>0.3518<br>0.1439	ANOVA	ns		Normality cannot be discarded	Homoscedasticity cannot be discarded	
	% CD11c+ CD8+ cells	A<br>B<br>C	PBS<br>5FU TSf-ISPA<br>double 5FU TSf-ISPA	0.128<br>0.1058<br>0.182	0.03564<br>0.03079<br>0.05263	ANOVA	0.0325	B vs C p-value 0.012	Normality cannot be discarded	Homoscedasticity cannot be discarded	
5	% CD11b+ Ly6G+ Ly6C+/low cells		5FU TSf-ISPA<br>double 5FU TSf-ISPA	20.1<br>20.23	5.519<br>3.49	t-test	ns		Normality cannot be discarded	Homoscedasticity cannot be discarded	
	% CD11b+ Ly6C+ Ly6G- cells		5FU TSf-ISPA<br>double 5FU TSf-ISPA	3.468<br>2.063	2.342<br>0.7166	t-test	ns		Normality cannot be discarded	Homoscedasticity cannot be discarded	

6	% CD11b+ Ly6G+ Ly6C+/low cells	A	NI	1.65	0.05	Kruskal-Wallis	0.0159	A vs B p-value 0.035<br>A vs C p-value 0.0084	Normality cannot be discarded		
		B	double 5FU TSf-ISPA	8.617	1.332						
		C	PBS	13.1	6.806						
	% CD11b+ Ly6G- Ly6C+ cells	A	NI	2.433	0.3512	Kruskal-Wallis	0.0092	A vs B p-value 0.0475<br>A vs C p-value 0.0055	Normality cannot be discarded		
		B	double 5FU TSf-ISPA	3.617	0.6735						
		C	PBS	5.3	2.252						
