# Supplementary figures and images for: Control of myeloid-derived suppressor cell dynamics potentiates vaccine protection in multiple mouse models of Trypanosoma cruzi infection
Source: Front Immunol. 2024 Nov 1;15:1484290. doi: 10.3389/fimmu.2024.1484290 (PMC11568482; doi:10.3389/fimmu.2024.1484290)

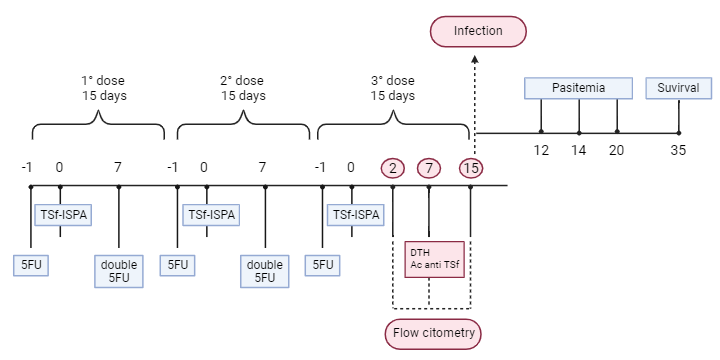

Supplement: Supplementary file 3 [file Image1.png]

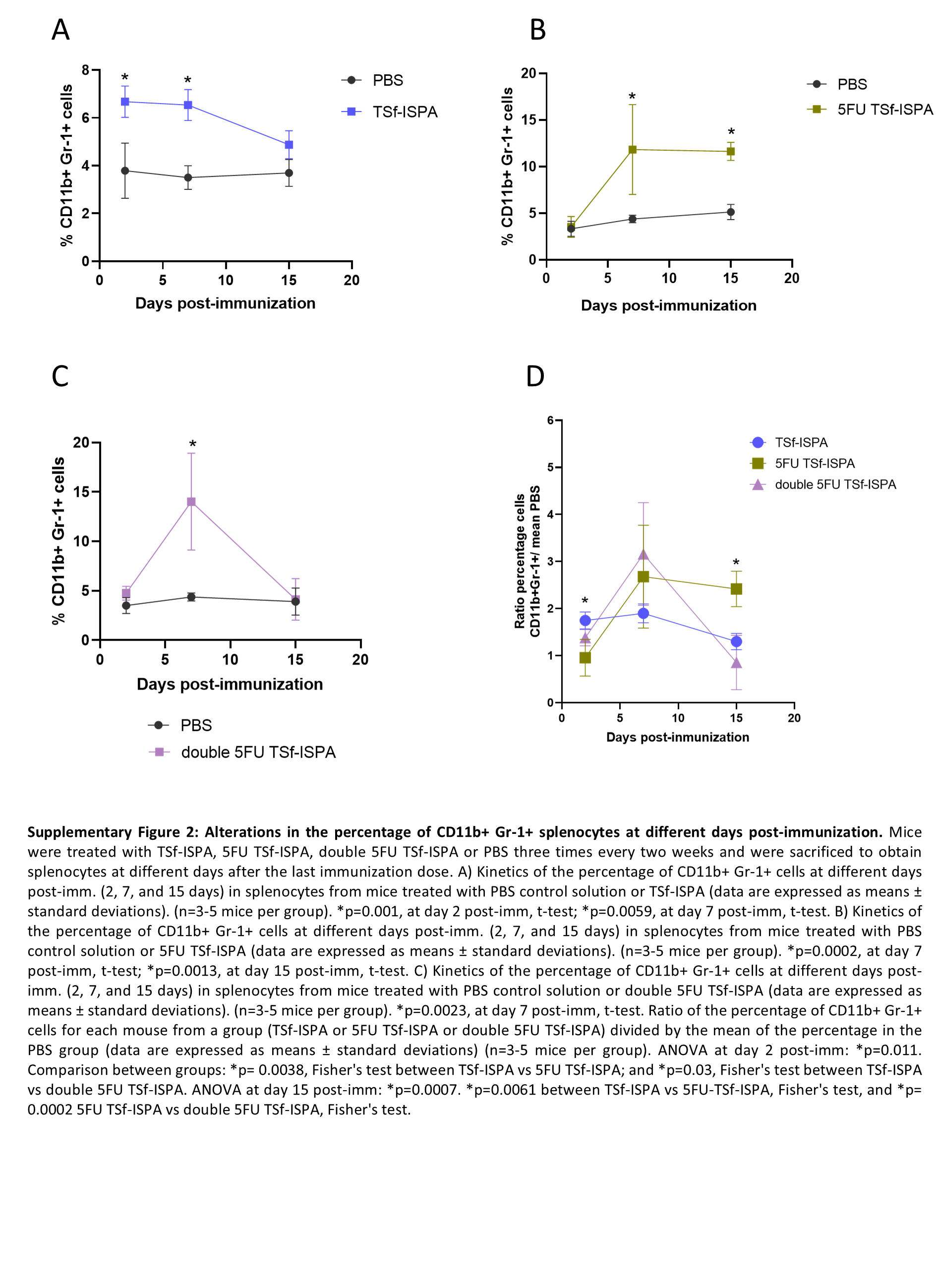

Supplement: Supplementary file 4 [file Image2.tif]

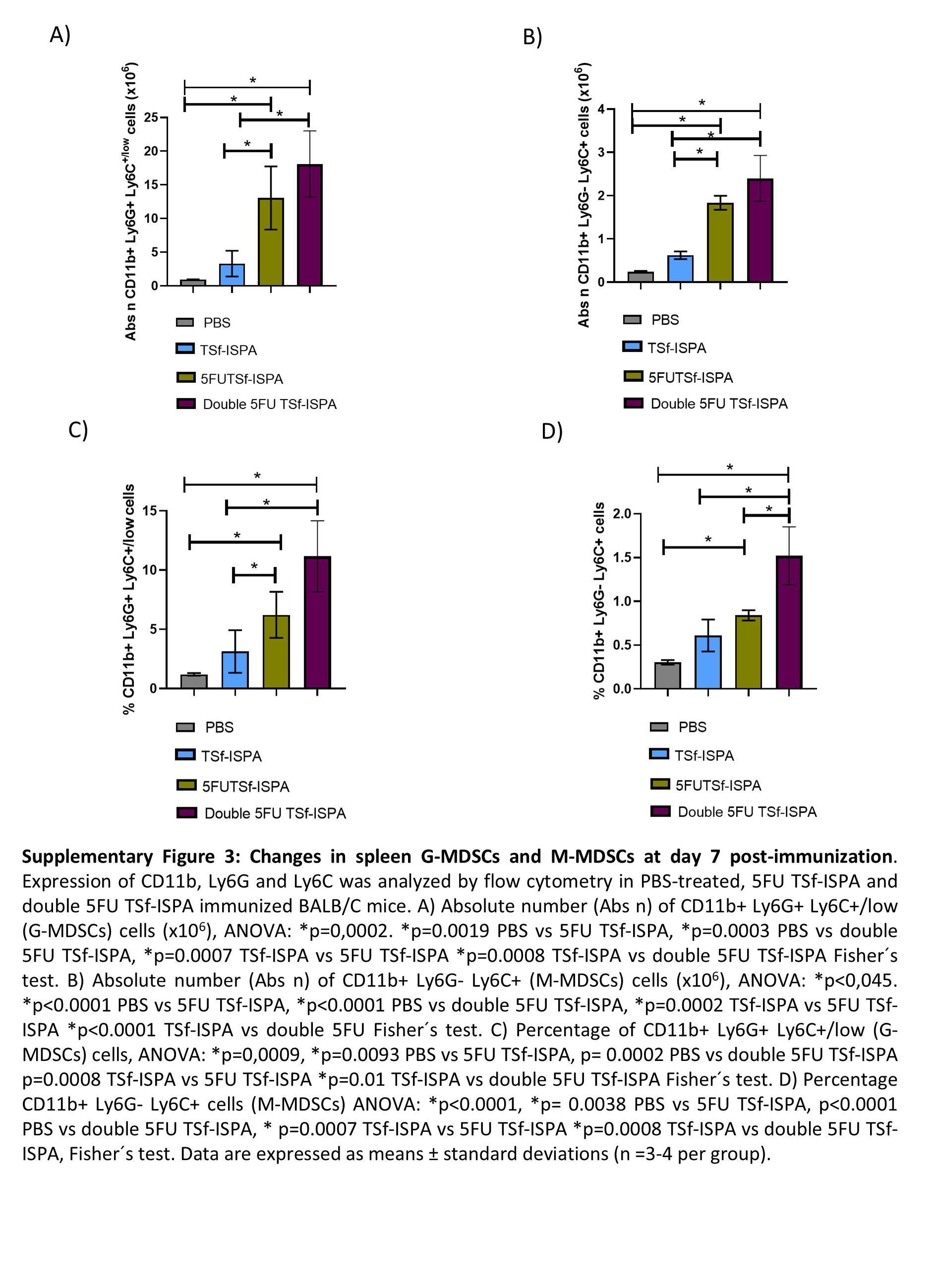

Supplement: Supplementary file 5 [file Image3.tif]

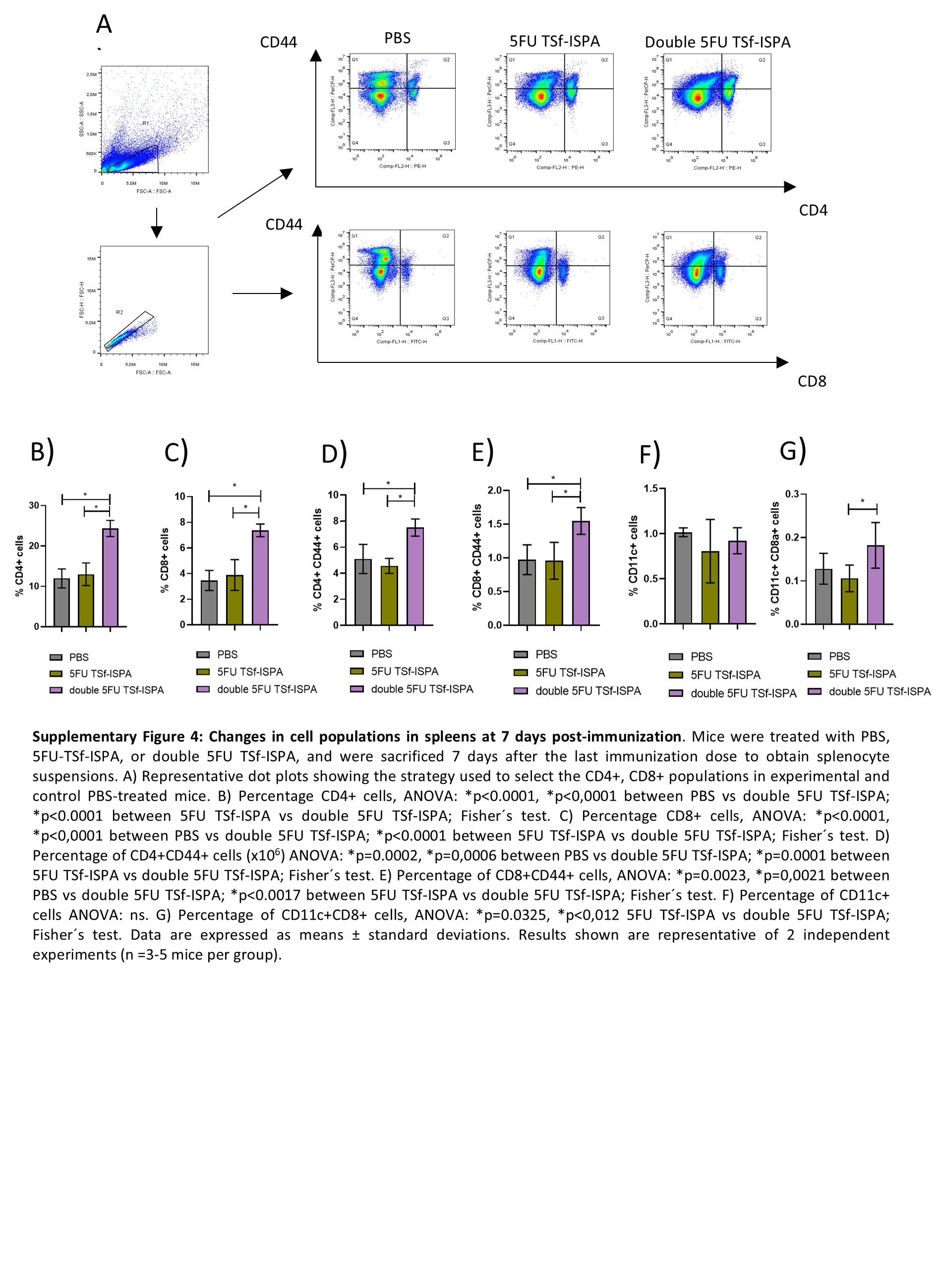

Supplement: Supplementary file 6 [file Image4.tif]

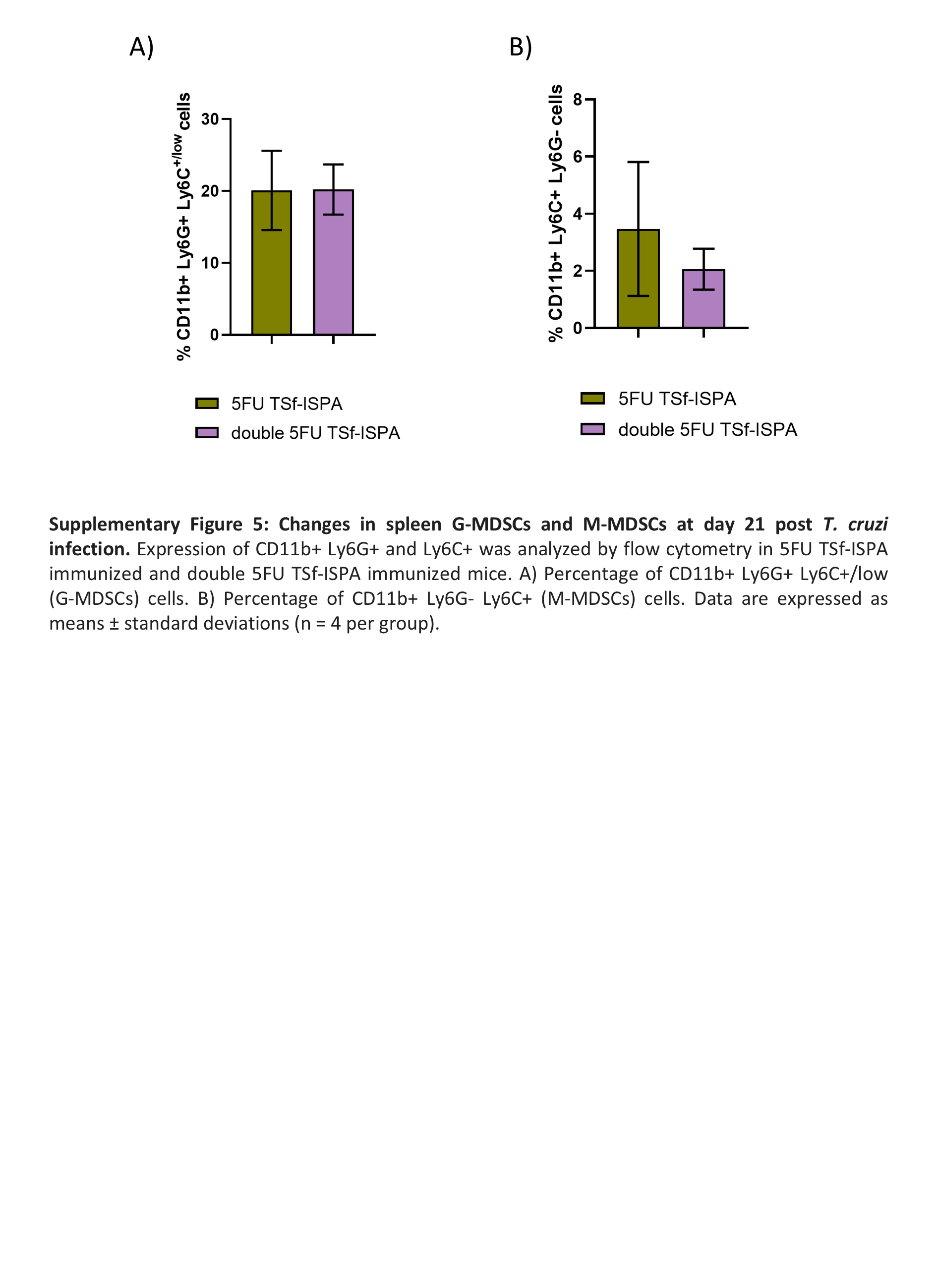

Supplement: Supplementary file 7 [file Image5.tif]

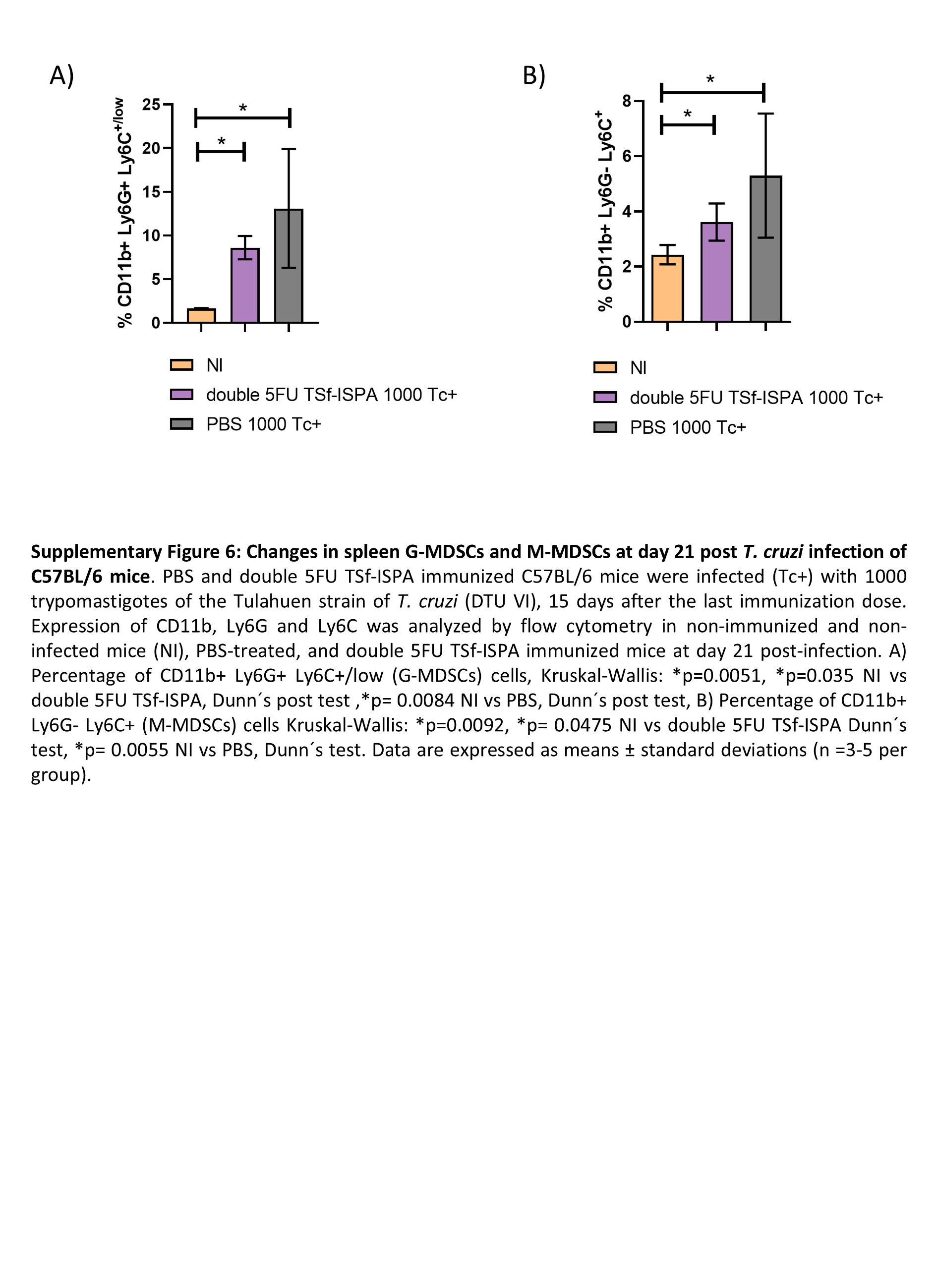

Supplement: Supplementary file 8 [file Image6.tif]

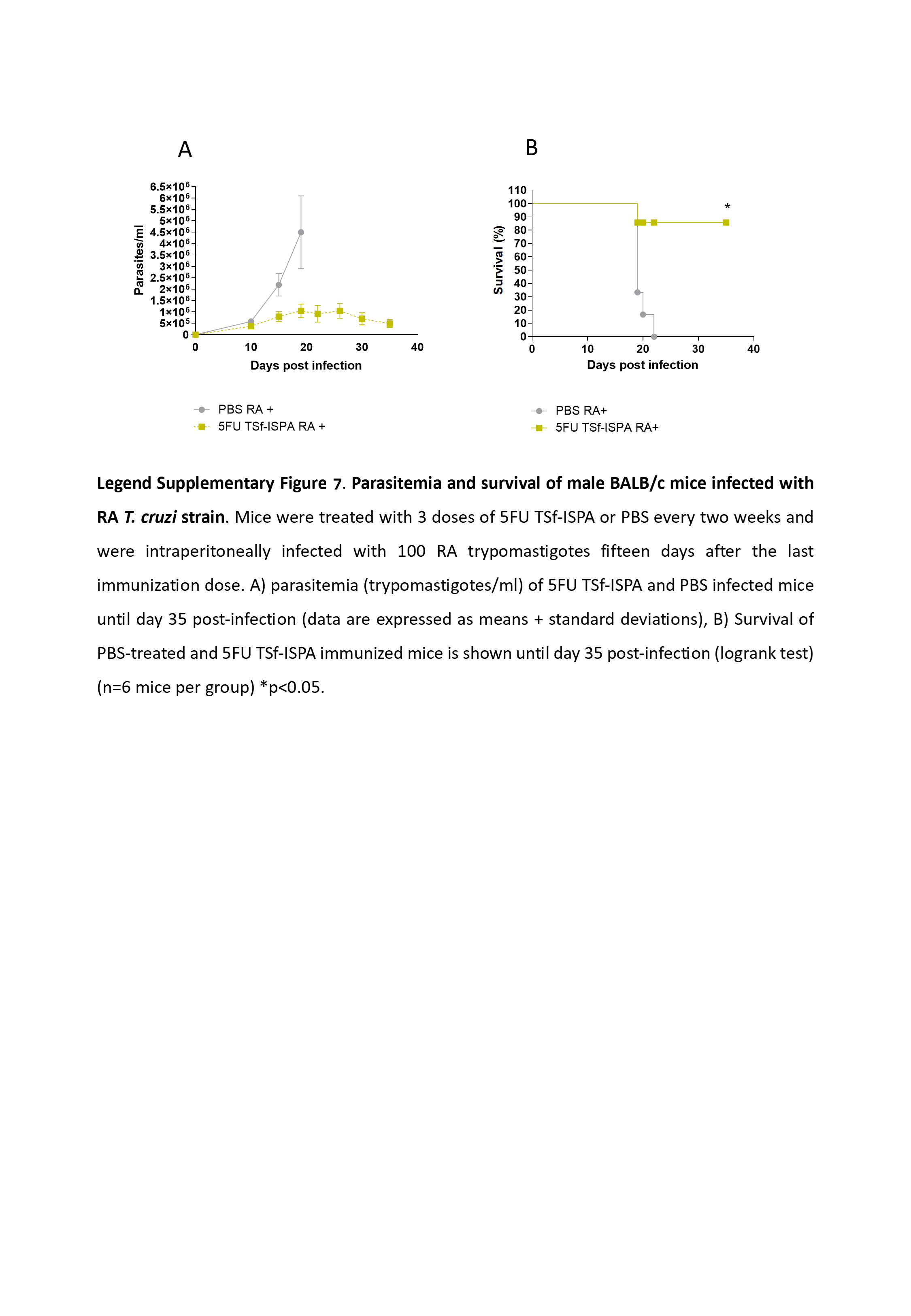

Supplement: Supplementary file 9 [file Image7.tif]
